# The Impact of Gamified Interventions on the Management of Chronic Obstructive Pulmonary Disease: Systematic Literature Review

**DOI:** 10.2196/69510

**Published:** 2025-05-30

**Authors:** Jinsong Chen, Tingzhong Yang, Qilian He, Mingli Pang, Ying Cao, Zheng Liu, Linfei Li, Hsing-I Liu, Christopher Bullen

**Affiliations:** 1 Department of Public Administration Law School Hangzhou City University Hangzhou China; 2 School of Public Affairs Zhejiang University Hangzhou China; 3 National Institute for Health Innovation University of Auckland Auckland New Zealand; 4 Research Center for Tobacco Control, Zhejiang University and Research Center for Digital Health Theory and Management, ZJU National Health Big Data Institute, Center for Tobacco Control Research Zhejiang University School of Medicine Zhejiang University Hangzhou China; 5 School of Nursing College of Medicine Dali University Dali China; 6 Tencent IEG Social Value Exploration Center Shenzhen China; 7 China Taiping Insurance Group Ltd. Shanghai China

**Keywords:** gamified interventions, chronic obstructive pulmonary disease, COPD management, digital health, patient engagement, pulmonary rehabilitation

## Abstract

**Background:**

Chronic obstructive pulmonary disease (COPD) requires consistent sustained management, including regular physical activity, pulmonary rehabilitation, and self-care adherence. Despite strong clinical guidelines, patient engagement remains a major challenge, leading to suboptimal disease control and increased health care use. Gamified interventions have emerged as potential tools to improve adherence, motivation, and outcomes in chronic disease management. However, their effectiveness and implementation in COPD remain underexplored.

**Objective:**

This review synthesizes current evidence on gamified interventions for COPD management to evaluate their effectiveness, focusing on patient engagement, physical outcomes, and quality of life.

**Methods:**

We conducted a systematic search in PubMed, Scopus, Web of Science, Embase, IEEE Xplore, Cochrane Library, and China National Knowledge Infrastructure for studies published from January 2014 to October 2024. Only original studies involving trials (both randomized controlled trials [RCTs] and non-RCTs), intervention studies, feasibility studies, cross-sectional surveys, or qualitative studies were included.

**Results:**

A total of 29 studies met the inclusion criteria: 11 (38%) RCTs; 7 (24%) pilot studies; 5 (17%) observational studies (including qualitative studies); and 6 (21%) other studies using gamified technologies such as virtual reality, exergames, and mobile apps. Compared to nongamified methods, gamified interventions provided an engaging, home-based alternative for COPD management, supporting long-term rehabilitation. Gamification features such as real-time feedback, adaptive challenges, and personalized goals increased patient adherence and motivation, with high engagement seen in virtual reality and exergame-based interventions, and showed notable improvements in COPD management, enhancing exercise tolerance, self-management, and symptom control. However, most of the studies (22/29, 76%) were of short duration, with small sample sizes.

**Conclusions:**

Gamified COPD management tools offer flexibility and empower patients to self-manage their condition, potentially reducing the need for clinic visits. Gamified interventions show promise in COPD management, although current studies have methodological limitations. Future research should focus on conducting larger trials to assess the sustained impact of gamified interventions on COPD outcomes; developing culturally relevant adaptations to enhance the global applicability of these interventions; and collaborating with patients, clinicians, and game developers to make the interventions more engaging and effective.

## Introduction

### Background

Chronic obstructive pulmonary disease (COPD) is a progressive, irreversible respiratory condition characterized by persistent airflow limitation, which collectively reduces patients’ physical capabilities and impairs their quality of life (QoL). COPD affects >300 million individuals worldwide and ranks as the third leading cause of death, underscoring an urgent need for innovative, accessible management solutions [1]. COPD management typically focuses on symptom alleviation and preventing disease progression through pulmonary rehabilitation (PR; defined as a multidisciplinary, evidence-based intervention for chronic respiratory diseases such as COPD, combining exercise training, education, and behavioral therapy to reduce symptoms, improve physical and psychological well-being, and enhance long-term health behaviors), pharmacological interventions, and behavior modification (such as smoking cessation and dietary change) [1]. However, adherence to these programs is often low, due in part to a lack of patient engagement, access barriers, and the need for long-term ongoing use of the interventions [2]. Recent advances in digital health, particularly gamified interventions, show promise to enhance adherence and self-management in patients with COPD by incorporating engaging, game-like elements into COPD management programs.

Gamification in health care, defined as using game design elements in nongame contexts, is emerging as a valuable tool in managing chronic diseases, particularly in PR and home-based COPD management [3]. Gamified interventions leverage mechanisms such as rewards, competitive elements, real-time feedback, and immersive virtual environments to enhance motivation; foster active participation; and sustain adherence, particularly to exercise-focused regimens [4]. Recent systematic reviews indicate that gamified eHealth interventions can improve physical activity levels in adults with chronic diseases and significantly enhance physical outcomes, adherence, and QoL [1,5]. In COPD, where symptoms often lead to a sedentary lifestyle, these elements can provide a sense of accomplishment and motivation, addressing common barriers to physical activity and rehabilitation.

A systematic review by Chang et al [5] highlighted the potential of gamified interventions to enhance exercise endurance and QoL among patients with COPD, especially those incorporating telemonitoring, web-based tools, and virtual reality (VR). VR as a vehicle for PR has a growing evidence base; for example, Liu et al [2] conducted a meta-analysis to explore VR’s role in COPD management and found that VR-assisted PR can improve lung function and exercise capacity. By making exercise engaging and immersive, delivery via VR potentially mitigates the monotony associated with traditional exercise–focused PR, encouraging regular participation and improving outcomes. In addition, a systematic review and meta-analysis by Obrero-Gaitán et al [3] found supportive evidence for the effectiveness of VR-based PR in COPD, reporting significant improvements in physical performance and mobility.

Such immersive, game-like platforms not only aid in physical rehabilitation but also enhance patient engagement. In a scoping review, Dalko et al [6] found supportive evidence for VR in rehabilitation for patients recovering from post–COVID-19 condition who had chronic respiratory problems [6]. These findings support the idea that VR’s engaging nature may appeal to patients with COPD facing similar challenges of motivation and compliance.

### Objectives

While these studies found promising results, most gamified interventions are in the early stages of development. As the recent systematic reviews highlight, there is a need for larger, long-term rigorous studies to validate their effectiveness, cost-effectiveness, acceptability, and sustained use for different populations. This review synthesizes existing literature to assess the impact of gamified interventions on COPD management and PR, focusing not only on physical outcomes and QoL improvements but also on engagement and adherence. By consolidating these findings, this study aims to provide a comprehensive understanding of gamification’s potential role in enhancing care for patients with COPD.

## Methods

### Overview

The primary objective of this study was to systematically review and evaluate the impact of gamified interventions on the management of COPD, including PR. Specifically, this study aimed to assess how gamification influences key outcomes in COPD management, such as patient adherence to treatment regimens, physical activity, symptom management, and QoL. In addition, we sought to assess the accessibility, usability, and patient satisfaction associated with these interventions, considering whether gamification supports long-term behavior change and self-management. By synthesizing findings from recent studies, this study aimed to provide valuable insights into the current evidence, limitations, and the potential of gamified approaches in enhancing COPD care, as well as to offer recommendations for future research directions in this emerging area of digital health.

### Search Strategy

The search strategy targeted studies on gamified interventions for COPD management, emphasizing recent advancements over the past 10 years (January 2014-October 2024) to maintain relevance in this rapidly evolving field. We searched 7 databases—PubMed, Scopus, Web of Science, Embase, IEEE Xplore, Cochrane Library, and China National Knowledge Infrastructure—capturing a broad range of research in biomedical and digital health.

The search terms in Table 1 were identified for 3 domains: “chronic obstructive pulmonary disease,” “gamification,” and “condition management” as well as “rehabilitation,” with terms within each domain connected by the Boolean operator “OR” and domains linked by “AND.” Detailed search strategies for each database are shown in Multimedia Appendix 1.

The searches included studies published in English and Chinese to capture a more global perspective than reviews relying solely on English-language databases. Boolean operators were used to ensure comprehensive coverage, and all retrieved studies were imported into EndNote 20 (Clarivate) to facilitate screening, deduplication, and data extraction.

**Table 1:** Search domains and terms.

Domains	Search terms
Chronic obstructive pulmonary disease	“Chronic Obstruct* Pulmon* Disease” OR “COPD” OR “Pulmon* Disease, Chronic Obstruct*” OR “Chronic Bronchit*” OR “Emphysem*” OR “Respirator* Disease” OR “Pulmon* Disorder”
Gamification	“Gamif*” OR “Game-based” OR “Serious Game*” OR “Gaming” OR “Digital Game*” OR “Health Game*” OR “Game Mechanic*” OR “Mobile Game*” OR “Interactive Game*” OR “Exergame*” OR “Behavioral Gamif*” OR “Virtual Reality” OR “Augmented Reality”
Condition management	“Self-Manage*” OR “Self-Care” OR “Disease Manage*” OR “Chronic Disease Manage*” OR “Symptom Monitor*” OR “Pulmon* Rehab*” OR “Pulmon* Rehabilitat*” OR “Exacerbat* Prevent*” OR “Patient Engage*” OR “Patient Adher*” OR “Treat* Adher*” OR “Health Monitor*” OR “Behavior* Change” OR “Behaviour* Change” OR “Condition Manage*” OR “Clinical Monitor*” OR “Intervent* Manage*”

### Inclusion and Exclusion Criteria

Inclusion criteria were as follows: (1) languages—studies published in English and Chinese were included to capture a breadth of global research; (2) duration—publications from the last 10 years (January 2014-October 2024) were included to reflect recent advances in gamification and digital health; and (3) study types—only original studies involving trials (both randomized controlled trials [RCTs] and non-RCTs), intervention studies, feasibility studies, cross-sectional surveys, or qualitative studies were included. Studies published solely as protocols or conference proceedings were excluded due to limited information on outcomes and intervention efficacy. Studies that did not incorporate gamification elements and those not addressing COPD as the primary health condition were also excluded.

### Data Extraction and Analysis

Data extraction and analysis were conducted in accordance with the PRISMA (Preferred Reporting Items for Systematic Reviews and Meta-Analyses) 2020 guidelines to ensure thorough and transparent reporting [7]. The PRISMA checklist is presented in Multimedia Appendix 2. Using a standardized data extraction form, 2 independent reviewers extracted relevant information from each study, reducing the risk of bias and enhancing data reliability. Key data collected included study design, sample size, population characteristics, intervention details (such as the type of gamification, platform, and duration), outcomes (eg, physical function, adherence, engagement metrics, and QoL), and any limitations reported. Multimedia Appendix 3 provides the template used for the data extraction and analysis form. Data on primary and secondary outcomes—such as physical activity levels, lung function, and QoL metrics—were specifically targeted to assess the effectiveness of gamified interventions in COPD management. A third reviewer addressed any discrepancies between the 2 primary reviewers. Once extracted, data were tabulated and summarized to facilitate cross-study comparisons. Given the diversity in gamification strategies and outcome measures, a narrative synthesis approach was used to identify common themes, potential correlations, and patterns across studies. Each study’s methodological quality and risk of bias were assessed using criteria aligned with the PRISMA guidelines. The risk-of-bias assessment was conducted using a qualitative approach, given the diverse study designs (both RCTs and non-RCTs) included in this review. For each study, the review evaluated methodological limitations and potential sources of bias, including risk-of-bias assessment, risk-of-bias findings, and limitations. The assessment framework considered several key domains that could potentially influence study validity: randomization procedures (eg, control group presence and characteristics, as well as blinding methods), sample characteristics (eg, sample size adequacy, dropout rates, and selection procedures), and implementation quality (eg, study duration, follow-up assessment, and intervention adherence). On the basis of these criteria, studies were classified as low risk, moderate risk, or high risk.

## Results

### Summary of Included Studies

**Figure 1:**
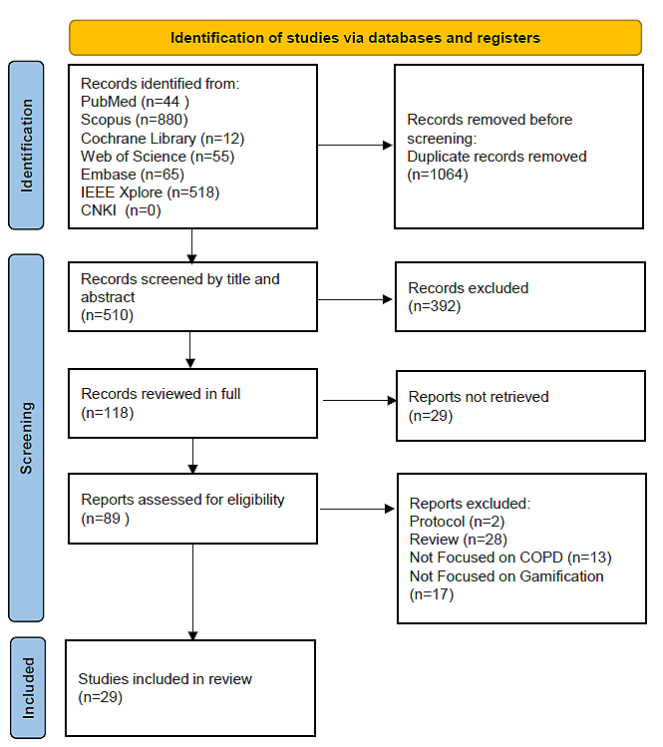
PRISMA (Preferred Reporting Items for Systematic Reviews and Meta-Analyses) flow diagram showing the number of studies identified, screened, assessed for eligibility, and included in the final analysis. CNKI: China National Knowledge Infrastructure; COPD: chronic obstructive pulmonary disease.

The study identified 29 studies published between January 2014 and October 2024 that investigated the impact of gamified interventions on COPD management. The studies examined a range of gamification approaches, including VR-based rehabilitation, mobile apps, and digital games designed to enhance patient engagement, physical activity, or symptom management. Multimedia Appendix 4 [8-36] provides a detailed summary of the data extraction and analysis for each included study. The PRISMA flowchart (Figure 1) outlines the study selection process.

### Study Designs and Settings

Most of the studies (10/29, 35%) were RCTs, reflecting the focus on evaluating intervention effectiveness [8,9]. Sample sizes ranged from small-scale feasibility trials with as few as 4 participants to larger studies with up to 106 participants [10,11]. Intervention durations varied substantially from single sessions to long-term studies lasting more than a year. Settings varied widely, including clinical, home-based, and hybrid models, with home-based approaches being common for virtual and gamified interventions [12,13].

Table 2 provides a summary of the study design, setting, sample size, and intervention duration for each reviewed study, capturing the diversity in research approaches across this field.

**Table 2:** Overview of study designs and settings.

Study	Study design	Setting	Sample size, n	Intervention duration
Tabak et al [8], 2014	Pilot RCT^a^	Hospital and primary care physiotherapy practices, Enschede, Netherlands	29	9 mo
Mazzoleni et al [14], 2014	RCT	Auxilium Vitae Rehabilitation Center, Volterra, Italy	40	3 wk
Kotrach et al [15], 2015	Pilot RCT	Mount Sinai Hospital, Montreal, Canada	12	3-6 h of individualized training, with follow-up at home
Hoaas et al [12], 2016	Mixed methods pilot study	Home-based with telemonitoring, Norway	10	2 y
LeGear et al [16], 2016	Randomized, within-participant crossover trial	St. Paul’s Hospital, Vancouver, Canada	10	Single session with two 15-min exercise interventions
Liu et al [17], 2016	Cross-sectional observational study	CIRO, Horn, Netherlands	61	—^b^
Bamidis et al [18], 2017	Multilevel intervention project	EU^c^-funded multisite study	Data not explicitly provided (ongoing multicenter trials)	3 y
Burkow et al [19], 2018	Feasibility study	Virtual group-based home setting	10	6 wk
De Las Heras et al [20], 2018	Qualitative study with focus groups and semistructured interviews	Aarhus University Hospital, Denmark; and Oulu University Hospital, Finland	13	—
Parent et al [21], 2018	Pilot feasibility study	Supervised hospital setting in Montreal, Canada	14	Single 30-min session
Rutkowski et al [22], 2019	RCT	Hospital-based pulmonary rehabilitation (stationary)	68	14 d
Sutanto et al [23], 2019	RCT	Outpatient clinic in Dr Moewardi Hospital, Surakarta, Indonesia	23	6 wk
Jung et al [13], 2020	Mixed methods study	Home-based rehabilitation, South and West Cumbria, United Kingdom	10	8 wk
Rutkowski et al [11], 2020	RCT	Specialist hospital in Głuchołazy, Poland	106	2 wk
Tu et al [24], 2020	Pilot feasibility study	Home-based (in-laboratory demonstration using smartwatch, smartphone, and VR^d^ headset)	Proof-of-concept demonstration; no specific patient sample size indicated	Demonstration sessions lasted 2-5 min
Rutkowski et al [9], 2021	RCT	Specialist hospital in Głuchołazy, Poland	50	2 wk (10 VR sessions)
Simmich et al [25], 2021	Qualitative study with semistructured interviews	Pulmonary support groups in Brisbane, Australia	19	NR (single interview session per participant)
Simmich et al [26], 2021	Pilot RCT	Home-based intervention, Brisbane, Australia	18	3 wk
Baxter et al [27], 2022	Mixed methods randomized usability study	Urban locations in southeast Queensland, Australia	24	Single session (3 inspirations using each device)
Oberschmidt et al [28], 2022	Qualitative study using interviews	Physiotherapy office in the Netherlands	7	6 mo
Finkelstein et al [29], 2023	Mixed methods randomized usability study	Icahn School of Medicine at Mount Sinai, New York, United States	9	Single session of using the VR app
Gabriel et al [30], 2023	Mixed methods quantitative and qualitative assessments	Icahn School of Medicine at Mount Sinai, New York, United States	18	Single session using the VR app
Gabriel et al [31], 2023	Qualitative study with semistructured interviews	Home-based, conducted virtually	9	Single session
Pancini et al [32], 2023	RCT	IRCCS INRCA, Italy	Not reported (study is in planning phase)	2 wk (four 20-min sessions)
Pardos et al [33], 2023	Methodology and pilot design	Remote care platforms for chronic disease management	Pilot system; no specific patient sample provided	—
Colombo et al [34], 2024	Single-group pilot study	In-hospital rehabilitation program, Italy	14	2 wk, with 20-min sessions, twice daily
Jin et al [35], 2024	RCT	The First Affiliated Hospital of Soochow University, China	80	6 wk
Kizmaz et al [36], 2024	RCT	Hospitalized patients at Pamukkale University Health Research and Application Centre, Turkey	50	Until discharge from hospital
McAnirlin et al [10], 2024	Proof of concept, mixed methods	Participants’ homes in upstate South Carolina, United States	4	4 mo

^a^RCT: randomized controlled trial.

^b^Not applicable.

^c^EU: European Union.

^d^VR: virtual reality.

### Types and Characteristics of Gamified Interventions

The reviewed studies applied various gamified interventions to support COPD management, ranging from VR programs to wearable activity trackers. Platforms included VR headsets, gaming consoles, and smartphone apps, each incorporating gamification elements such as motivational cues, real-time feedback, and rewards for progress [8,14,15]; for example, the Condition Coach COPD telehealth program used wearable devices and daily feedback tailored by physiotherapists [8], while BreathCoach provided real-time biofeedback for respiratory exercises [24].

Several interventions offered individualized tailoring to enhance relevance and usability, adapting exercise intensity or integrating patient preferences into their design. Integration with health care systems varied, with some interventions offering telemonitoring and health care provider supervision, such as the SmokeFreeBrain project [18]. Table 3 shows the details of the interventions, the platform, game elements, details of tailoring if included, and integration with health care.

**Table 3:** Summary of intervention characteristics.

Study	Intervention	Type	Platform or technology used	Game elements	Tailoring	Integration with health care
Tabak et al [8], 2014	Condition Coach COPD telehealth program	Motivational cues	Wearable device, smartphone app, and web-based portal	Motivational cues and daily feedback	Exercise schemes tailored by physiotherapists	Integrated with primary and secondary care; health care professionals monitored progress
Mazzoleni et al [14], 2014	Exergaming with Nintendo Wii	Exergames using Nintendo Wii Fit Plus	Nintendo Wii and Wii Balance Board	Real-time feedback and visual and auditory cues	Exercise intensity adjusted by physiotherapist	Supervised by health care professionals
Kotrach et al [15], 2015	Virtual game system for home exercise	Exergames using Nintendo Wii Fit U	Nintendo Wii Fit U	11 prevalidated games, with real-time feedback on performance	Individualized training sessions based on patient needs	Monitored by physiotherapists during in-hospital and home training
Hoaas et al [12], 2016	Telerehabilitation program	Telerehabilitation via self-management and telemonitoring	Treadmill, pulse oximeter, and iPad with a web page for telemonitoring	Regular feedback and remote support	Individually tailored exercise program	Weekly videoconferencing sessions with a physiotherapist
LeGear et al [16], 2016	Nintendo Wii-based exercise	Exergame using EA Sports Active on Nintendo Wii	Nintendo Wii with SenseWear Armband	Physical activities, including marching, dancing, and punching	Exercise intensity adjusted based on perceived exertion	NR^a^
Liu et al [17], 2016	GRAIL^b^	NR; focus on GRAIL technology and 6MWT^c^	GRAIL, 3D motion capture, and VR^d^ treadmill	VR environment used to simulate walking conditions	Not personalized beyond adjusting walking pace	NR
Bamidis et al [18], 2017	SmokeFreeBrain	Smoking cessation intervention using gamified apps, mini-games, and social media	Gamification app, neurofeedback, social media, mobile SMS text messaging, and pharmacological interventions	Tasks, goals, achievements (eg, “Crushing cigarettes” game, breathing control, and exercise gamification)	Interventions customized by demographic and geographic needs	Public health campaigns, electronic health records, and open data integration
Burkow et al [19], 2018	Virtual group intervention for behavior change	Virtual group exercise with follow-along videos and exercise diaries	Tablet-based app	Virtual group status, rewards, and exercise diary	Self-chosen individual exercises	Peer group exercises combined with remote support
De Las Heras et al [20], 2018	AR^e^ glasses	Use of AR glasses	Laster WAVƎ AR glasses	Visual and audio guidance during exercises	Adjustable brightness, head fixation, and interface improvements	Remote communication and feedback
Parent et al [21], 2018	High-intensity active video game	High-intensity exergames	Kinect motion capture (Xbox One) and Shape Up game	Repetitive exercise mini-games	Adjustments to exercise difficulty	NR
Rutkowski et al [22], 2019	VR training program	Virtual rehabilitation using Kinect-based motion training	Xbox 360 Kinect system and Kinect Adventures! game	Avatar-based mini-games involving rafting, ball hitting, and roller coaster riding	VR exercises tailored for each patient	Integrated into a structured pulmonary rehabilitation program
Sutanto et al [23], 2019	Videogame-assisted exercise training	Wii Fit–based exercise training	Wii Fit balance board and television system	Yoga, strength training, aerobic exercises, feedback system, and virtual trainer	Individualized program with game duration, difficulty, and scores recorded	Integrated into a hospital-based outpatient exercise program
Jung et al [13], 2020	VR pulmonary rehabilitation program	VR-supported pulmonary rehabilitation	VR headset (Pico Goblin) and pulmonary rehabilitation in VR app	3D avatars, educational modules, and immersive experience	Exercises tailored to severity level (MRC^f^ breathlessness scale level 4 or 5) of patient with COPD^g^	Real-time remote monitoring of heart rate and oxygen saturation
Rutkowski et al [11], 2020	VR rehabilitation for COPD	VR-based rehabilitation using Xbox Kinect and Kinect Adventures! software	Xbox 360, Kinect, and Kinect Adventures!	Mini-games involving rafting, ball hitting, dynamic balance, and coordination	Exercises adapted to patients’ abilities	Supervised by physiotherapists; heart rate monitored
Tu et al [24], 2020	BreathCoach	VR-assisted biofeedback breathing training using RSA-BT^h^	Smartphone, smartwatch (Empatica E4), and VR viewer (Google Cardboard)	Breathing control, interactive VR environments, avatars, and real-time biofeedback	Dynamic adjustment of breathing patterns based on real-time physiological data	NR
Rutkowski et al [9], 2021	Immersive VR therapy	Immersive VR therapy	VR TierOne device	Virtual therapeutic garden and metaphoric health recovery	Emotional balance recovery and mood improvement	Supervised by therapists
Simmich et al [25], 2021	Wearable technology and video games	AVGs^i^	Wearable activity trackers, smartphones, and AVGs such as Wii and Xbox Kinect	AVGs and wearable trackers	NR	Data shared with clinicians for feedback and improved clinical care
Simmich et al [26], 2021	AVG for physical activity	AVG focusing on physical activities	Smartphone app with Fitbit integration	Single-player and multiplayer modes, progress tracking, and rewards for completing exercises	Players selected difficulty levels for each exercise	Clinicians monitored progress via a web interface
Baxter et al [27], 2022	Virtual respiratory therapy	Virtual incentive spirometry via smartphone app	QUT Inspire app and smartphone	Visual rewards, breath timer, and inspiration counter	Adjustable microphone sensitivity and text or video instructions	NR
Oberschmidt et al [28], 2022	Exergame for COPD treatment	Exergame used as part of physiotherapy treatment	Television screen with motion-sensing camera	Audiovisual feedback during exercises and score tracking	Feedback based on exercise accuracy and adjustable difficulty levels	Integrated into routine physiotherapy treatment
Finkelstein et al [29], 2023	VR app for pulmonary rehabilitation	VR educational app	Oculus Quest 2	Interactive educational modules, MCQs^j^, and visual feedback	Simplified user interface with preset controls for ease of use	NR
Gabriel et al [30], 2023	VR-based system for pulmonary rehabilitation	VR-based system for pulmonary rehabilitation	Oculus Quest 2	Educational modules, MCQs, and guided exercises	Simplified interface, single-button navigation, and custom instructions	NR
Gabriel et al [31], 2023	VR-based system for pulmonary rehabilitation	VR-based exercise app for pulmonary rehabilitation	VR headset and controllers	Interactive guided exercises and visual feedback	Simplified controls and visual guidance for exercises	Focused on self-management
Pancini et al [32], 2023	Overcoming COPD	VR-based relaxation combined with savoring strategies	VR headset with immersive natural scenarios	Narrated virtual walks, visual and audio feedback, and positive emotion amplification	Personalized savoring exercises	Incorporated into standard pulmonary rehabilitation
Pardos et al [33], 2023	Remote monitoring and gamification platform	Personalized coaching with exergames and mental health games	Smartphone app, smartwatches, and Bluetooth-enabled devices	Credit-based system, scores, rewards, and health recommendations	Customized recommendations	Integration with PHR^k^ and third-party apps
Colombo et al [34], 2024	VR endurance training	Semi-immersive VR cycling in a virtual park environment	Cycle ergometer, pulse oximeter, and wide screen projection	Visual feedback, real-time cycling metrics, and first-person navigation	Exercise intensity based on baseline conditions	Continuous supervision by physiotherapists
Jin et al [35], 2024	Somatosensory interactive game	Somatosensory interactive games involving arm movements for exercise	Motion-based games: Kitchen Sharp Knife, Swimming Master, and Table Tennis Master	Real-time visual feedback and engaging tasks	Patients adjusted exercise based on comfort level	Integrated with physiotherapist-supervised pulmonary rehabilitation programs
Kizmaz et al [36], 2024	VR for COPD exacerbation	VR cycling simulation in the forest combined with pulmonary rehabilitation	Oculus Quest 2	Immersive cycling simulation in a forest environment	NR	Integrated with pulmonary rehabilitation sessions, supervised by physiotherapists
McAnirlin et al [10], 2024	Nature-based VR experiences	Nature-based VR experiences	Oculus Quest 2	Cocreated 360-degree videos of personalized, nature-based scenes	Personalized VR based on participants’ outdoor memories	NR

^a^NR: not reported.

^b^GRAIL: Gait Real-time Analysis Interactive Lab.

^c^6MWT: 6-minute walk test.

^d^VR: virtual reality.

^e^AR: augmented reality.

^f^MRC: Medical Research Council.

^g^COPD: chronic obstructive pulmonary disease.

^h^RSA-BT: respiratory sinus arrhythmia biofeedback-based breathing training.

^i^AVG: active video game.

^j^MCQ: multiple-choice question.

^k^PHR: personal health record.

### Effectiveness of Gamified Interventions

Gamified interventions showed positive impacts on COPD management outcomes, particularly in improving exercise tolerance and physical fitness; for instance, Mazzoleni et al [14] found a significant increase in 6-minute walk test distance among participants using exergames, with the experimental group improving by 97.4 meters. Similarly, Rutkowski et al [11] observed increased exercise performance in the VR groups compared to the traditional exercise group, as shown in Table 4.

Regarding behavioral outcomes, gamified interventions were effective in increasing motivation and adherence to exercise; for example, LeGear et al [16] noted high participant enjoyment and engagement with Wii-based exergames, enhancing exercise adherence. Furthermore, Hoaas et al [12] reported long-term adherence to telerehabilitation, showing improved self-management and coping skills among patients with COPD.

QoL improvements were also observed, with several interventions enhancing emotional well-being and reducing anxiety; for example, Pancini et al [32] reported reduced anxiety and stress in participants using VR relaxation interventions, contributing to overall QoL. Such findings suggest that gamified approaches can not only support physical health but also promote psychological resilience and well-being in patients with COPD [8,13].

**Table 4:** Effectiveness of gamified chronic obstructive pulmonary disease (COPD) interventions.

Study	Effectiveness results	Behavioral outcomes	QoL^a^ improvements
Tabak et al [8], 2014	Exacerbations: telehealth group=33 (median 2.0, IQR 1.0-3.0); hospitalizations: telehealth group=4 (median 5.5, IQR 4.8-6.3) d, CG^b^=5 (median 7.0, IQR 6.0-7.0) d; QoL (EQ-5D VAS^c^): telehealth group=72.3, CG=62.4; no statistically significant difference in clinical outcomes between groups	Improved self-management of exacerbations (86.4% diary adherence)	EQ-5D VAS score: telehealth group—from 64.7 (baseline) to 72.3 (3 mo); CG—from 65.0 (baseline) to 62.4 (3 mo); CCQ^d^ score: telehealth group—from 2.0 (baseline) to 1.8 (3 mo)
Mazzoleni et al [14], 2014	6MWT^e^: EG^f^=+97.4 m, CG=+61.1 m (*P*=.03); TDI^g^ score: EG=3.9, CG=2.2 (*P*<.001); SGRQ^h^ score: EG=−10.8, CG=−12.7 (*P*=.66); significant improvement in 6MWT and dyspnea for EG compared to CG	Improved patient motivation and engagement in EG	SGRQ score improved
Kotrach et al [15], 2015	Exercise tolerance (6MWD^i^), heart rate, and oxygen saturation monitored; mean 6MWD was 306 (SD 81) m at baseline; preliminary results showed that participants could maintain exercise training after PR^j^ using VGS^k^	Dyspnea and leg discomfort increased, indicating exertion during exercise	Not reported in the preliminary findings
Hoaas et al [12], 2016	Average adherence: 43.3% for daily diary, 56.2% for exercise training; no dropouts; long-term adherence despite motivational challenges	Participants reported better self-management and coping with COPD	Reported improved health and increased capacity for daily activities
LeGear et al [16], 2016	Energy expenditure: Wii group (mean 353.5, SD 134.1 J) vs treadmill group (mean 317.1, SD 105.2 J), mean difference 36.3 J (95% CI 31.4 to 104); heart rate: Wii group (mean 112.5, SD 13.2 bpm) vs treadmill group (mean 112.7, SD 10.2 bpm), mean difference −0.167 (95% CI −4.83 to 4.50); no significant difference in energy expenditure, heart rate, or perceived exertion between Wii and treadmill groups	Participants reported enjoyment and perceived feasibility of Wii exercises at home	Not specifically reported in this study
Liu et al [17], 2016	Patients with COPD walked 27.5 m less on GRAIL^l^ vs overground 6MWT (*P*<.001); healthy older adults walked 23.6 m more on GRAIL (*P*<.001); GRAIL showed good reproducibility for both groups: ICC^m^ of 0.80 for patients with COPD (95% CI 0.61 to 0.89) and 0.65 for healthy older adults (95% CI 0.05 to 0.86)	Improved reproducibility and patient engagement with virtual environment for patients with COPD	No QoL data reported
Bamidis et al [18], 2017	Efficacy of PSAs^n^, e-cigarette interventions, and neurofeedback protocols; expected positive impacts on reducing smoking among groups considered high risk	Increased adherence to smoking cessation interventions using gamification and ICT^o^	Expected improvements in smoking-related morbidity and mortality rates
Burkow et al [19], 2018	Increase in physical activity from 2.9 to 5.9 sessions per wk during the program; 77% adherence to group exercises	Positive impact on motivation to engage in physical activity	Improved well-being and mood reported
De Las Heras et al [20], 2018	Positive perception of AR^p^ glasses, particularly ease of use and exercise guidance; patients saw value in the AR glasses for telerehabilitation, although some found them heavy	Motivation to use AR glasses for physical exercise and rehabilitation	NR^q^
Parent et al [21], 2018	Peak minute ventilation (36.8 L/min in squatting game) and peak METs^r^ (4.4 in squatting game); high-intensity games met exercise guidelines; Borg scores for leg exertion (13-14)	High perceived enjoyment and willingness to engage in home-based rehabilitation	QoL not directly measured
Rutkowski et al [22], 2019	Improved physical fitness as measured by the SFT^s^; significant within-group improvements (*P*<.05) in SFT (sit and reach test: from 0.0 to 0.7, 6MWT: from 494.9 to 469.9)	VR^t^ group showed enhanced motivation and adherence	NR
Sutanto et al [23], 2019	6MWD, dyspnea (TDI), and health-related QoL (SGRQ); 6MWD improved significantly (EG—from 376.6 to 420.0 m; *P*<.001; CG—from 410.7 to 477.5 m; *P*<.001), without any difference between groups	NR	Significant SGRQ score reduction in both groups (EG—from 57.7 to 30.6; *P*<.05; CG—from 54.1 to 29.4; *P*<.05), without any difference between groups
Jung et al [13], 2020	Improved compliance, physical health (mobility and flexibility), and psychological well-being; significant improvement in patients’ physical function, along with reduced anxiety and depression	Increased confidence and motivation to exercise	Improved self-reported health-related QoL
Rutkowski et al [11], 2020	Significant improvement in SFT (arm curl, chair stand, and 6MWT; *P*<.05); ET^u^+VR superior to ET (eg, 6MWT: ET+VR=+39.11 m, ET=+16.24 m; *P*<.05)	Enhanced motivation and adherence in VR-based exercises	NR
Tu et al [24], 2020	Feasibility of smart in-home breathing training with RSA-BT^v^; real-time biofeedback effectively guided breathing patterns	Improved engagement with breathing exercises due to immersive VR	NR
Rutkowski et al [9], 2021	Reduction in emotional tension (*P*<.001), external stress (*P*<.001), depression (*P*<.001), and anxiety (*P*<.001); VR group showed significant stress, anxiety, and depression reduction compared to CG	Increased mood and emotional balance through immersive therapy	Statistically significant improvements in psychological well-being
Simmich et al [25], 2021	Use of the game (58.6% of d logged), daily steps, and MVPA^w^; 9 min/d increase in MVPA (EG) and 2% decrease in steps (EG) vs 13% decrease (CG)	Positive correlation between game use and steps; weak correlation with MVPA	No significant improvements reported
Simmich et al [26], 2021	Perceptions of wearables and AVGs^x^ as tools for rehabilitation; participants found wearable trackers useful for quantifying activity, setting goals, and tracking improvements over time	AVGs were seen as fun and motivating for physical activity, but some participants felt that they were too difficult or not beneficial	No specific tools used to measure QoL, but general health benefits of physical activity were discussed
Baxter et al [27], 2022	Comparable inspiration durations between QUT Inspire (mean 7.3, SD 2.0 s) and Triflo II (mean 7.5, SD 2.3 s; *P*=.79); no significant differences in usability or performance between the app and the clinical device	Some users preferred app due to less perceived inspiratory effort	NR
Oberschmidt et al [28], 2022	Key patient values identified: independence, personal guidance, trust, and regularity; exergames supported values such as independence and challenge but hindered personal guidance and social interaction	Independence valued but personal guidance needed when using exergames	NR
Finkelstein et al [29], 2023	High usability and user acceptance (mean SUS^y^ score: 95.8); 89% of the participants successfully completed the first task, and 100% completed tasks 2 and 3 without prompts	High interest in using VR for patient empowerment and PR education	NR
Gabriel et al [30], 2023	High usability scores (SUS score: 95.8/100); successful completion of PR tasks by all participants with minimal guidance	Increased willingness to engage with home-based PR through VR	NR
Gabriel et al [31], 2023	High acceptability and usability of the VR-based system; increased motivation and engagement due to the novel, immersive approach	Positive feedback on ease of use and enjoyment of the exercises	NR
Pancini et al [32], 2023	Reduction in anxiety, depression, and stress; increased relaxation and emotional well-being; expected to improve emotional well-being (based on prior research with similar methods)	Participants expected to experience increased emotional resilience	Expected improvements in emotional and psychological well-being
Pardos et al [33], 2023	Development of personalized recommendations based on health data; early results show potential for increased adherence to care plans using personalized recommendations	Expected improvement in health-related behavior through gamification	NR
Colombo et al [34], 2024	Adherence rate of 85.71%; mean 6MWT distance improved to 520.50 (SD 69.24) m; significant improvements in exercise capacity (*P*<.05)	Increased motivation to exercise through VR	NR
Jin et al [35], 2024	Significant improvements in 6MWD and Brief-BESTest^z^ at 3 mo after the intervention (*P*<.001); EG maintained higher endurance and balance for 12 mo	Enhanced exercise tolerance and balance function; motivation sustained for 3 mo	Significant balance and exercise tolerance improvement
Kizmaz et al [36], 2024	Sit-to-stand test: significant improvement in PR+VR group (*P*<.001); COPD assessment test: significant reduction (*P*<.001), VR+PR group had greater improvements	Increased motivation and adherence to exercise reported in VR+PR group	Greater improvement in daily activities (London Chest Activity of Daily Living) in PR+VR group (*P*<.001)
McAnirlin et al [10], 2024	Psychological well-being, heart rate, respiratory rate, and oxygen saturation; positive changes in well-being and presence; no cybersickness reported	Participants experienced positive emotional responses, reflective of nostalgic memories	Reported feelings of autonomy, positive emotions linked to memories, and restorative effects

^a^QoL: quality of life.

^b^CG: control group.

^c^VAS: visual analog scale.

^d^CCQ: Clinical COPD Questionnaire.

^e^6MWT: 6-minute walk test.

^f^EG: experimental group.

^g^TDI: transition dyspnea index.

^h^SGRQ: St George’s Respiratory Questionnaire.

^i^6MWD: 6-minute walk distance.

^j^PR: pulmonary rehabilitation.

^k^VGS: virtual game systems.

^l^GRAIL: Gait Real-time Analysis Interactive Lab.

^m^ICC: intraclass correlation coefficient.

^n^PSA: public service announcement.

^o^ICT: information and communication technology.

^p^AR: augmented reality.

^q^NR: not reported.

^r^MET: metabolic equivalent of task.

^s^SFT: Senior Fitness Test.

^t^VR: virtual reality.

^u^ET: exercise training.

^v^RSA-BT: respiratory sinus arrhythmia biofeedback-based breathing training.

^w^MVPA: moderate to vigorous physical activity.

^x^AVG: active video game.

^y^SUS: System Usability Scale.

^z^Brief-BESTest: Brief Balance Evaluation Systems Test.

### Engagement, Satisfaction, and Usability

The reviewed studies demonstrated strong engagement and satisfaction among patients with COPD using gamified interventions, with many participants expressing enjoyment and adherence to the programs; for instance, studies involving VR and exergames, such as the one by Rutkowski et al [9], showed high engagement due to immersive environments and interactive features, leading to consistent participation. Satisfaction metrics varied, with studies such as the one by Baxter et al [27] noting that visual rewards and timers within apps were especially motivating.

Adaptations to improve usability included simplified interfaces and controls, particularly for older adults with limited technological experience [29,30]. However, technical challenges were present, such as synchronization issues in Fitbit devices [25] and discomfort from equipment such as VR headsets [31]. Cultural adaptations were minimal but occasionally tailored to suit specific populations, as seen in the studies by Pardos et al [33] and Colombo et al [34], which adapted VR for older adults with COPD.

Table 5 provides detailed insights into engagement, satisfaction, and technical adjustments across the reviewed studies.

**Table 5:** Engagement, satisfaction, and usability characteristics of gamified interventions.

Study	Engagement	Satisfaction	Cultural adaptation	Technical adaptation	Challenges
Tabak et al [8], 2014	Web portal use: 86.4% of d; exercise adherence: 21%; activity coach used for 299 d (132 d monitoring and 167 d feedback)	Satisfaction (CSQ-8^a^): telehealth group—26.4, CG^b^—30.4 (out of 32)	NR^c^	Wearable and web-based portal; no adaptation for other platforms	Technical issues with activity coach (eg, cycling accuracy); low exercise adherence (21%)
Mazzoleni et al [14], 2014	7 additional Wii Fit sessions for EG^d^; all completed	Satisfaction: EG—42.4, CG—43.9 (out of 49)	NR	None beyond Wii Fit system	Initial difficulty with balance board; exclusion of patients with motor limitations
Kotrach et al [15], 2015	All participants adhered to the VGS^e^ training	NR	NR	None beyond training sessions	Language barriers and patient ability to use VGS
Hoaas et al [12], 2016	On average, 3 diary entries per wk and 1.7 training sessions per wk	Increased self-efficacy and emotional safety; participants experienced health benefits	NRNR	iPad and treadmill used to adapt exercise training to home settings	Some technical difficulties with videoconferencing
LeGear et al [16], 2016	90% enjoyed Wii intervention, and 80% agreed that it could be used at home	NR	NR	None beyond standard Wii setup	Some participants required supervision for safe use
Liu et al [17], 2016	75% of patients with COPD^f^ and 90% of healthy older adults improved in second GRAIL^g^ test	NR	NR	No major technical adaptation beyond GRAIL VR^h^ setup	Complex setup required; difficulty for patients using self-paced treadmill
Bamidis et al [18], 2017	Various engagement tools: achievements and self-reported progress via mobile apps	NR	Tailored to socioeconomic and cultural contexts of various countries	Use of ICT^i^, mobile apps, SMS text messaging, and gamification across different platforms	Interoperability and customization for different health care systems; potential digital divide
Burkow et al [19], 2018	Peer monitoring and virtual group updates drove engagement	High acceptance; improved adherence to exercise routines; group motivation	NR	Tablet optimized with all other apps disabled	Minor technical issues (weather widget and activity sensor)
De Las Heras et al [20], 2018	High engagement; 12 out of 13 patients appreciated the AR^j^ glasses	Suggestions for improvement: adjustable screen, brightness, and head fixation	NR	Adjustments to AR glasses design and usability proposed by patients	Issues with head fixation during movement; brightness control
Parent et al [21], 2018	91% of the participants reached high-intensity levels in squatting exercises	Reported enjoyment, motivation for home use, and exercise tolerance	NR	None beyond Kinect customization	Participants experienced some discomfort in using new technology
Rutkowski et al [22], 2019	High adherence to both standard and virtual rehabilitation programs	Significant improvement in exercise tolerance	NR	Basic Kinect setup for stationary use; no advanced technical customizations	Minor technical issues with Kinect system
Sutanto et al [23], 2019	High adherence to the Wii Fit program	NR	Conducted in an Indonesian context but no specific cultural adaptations noted	Wii Fit program customized to the local setting; no major technical challenges	Limited intensity tracking, high cost of the Wii Fit program
Jung et al [13], 2020	High engagement due to enjoyment and immersive aspects	Improved QoL^k^, patient satisfaction, and engagement	NR	Feedback on improving headset weight and app functionality	Minor technical glitches; request for more customizable exercise levels
Rutkowski et al [11], 2020	High adherence (95% participation rate)	NR	NR	None beyond basic setup with Kinect	None significant; minor technical adjustments needed
Tu et al [24], 2020	High engagement in demonstration sessions; real-time feedback kept users on track	User feedback on usability, engagement, and real-time performance improvements	NR	Used lightweight algorithms and readily available devices for home use	Some technical refinements (eg, headset comfort and sound effects) suggested by users
Rutkowski et al [9], 2021	High engagement in VR group with full participation over the 2 wk	NR	NR	Use of VR TierOne device; simple immersion setup	NR
Simmich et al [25], 2021	Participants’ interest in wearables increased with social interaction and family involvement; challenges in long-term adherence were noted	Barriers and motivators for using wearables and AVGs^l^	NR	NR	Participants struggled with technological complexity and preferred more straightforward options
Simmich et al [26], 2021	High adherence to Fitbit (84.3% of d); moderate GEQ^m^ score of 30.4	Engagement metrics (IMI^n^ and GEQ); adherence to Fitbit	NR	No notifications, limiting engagement	Bluetooth synchronization issues with Fitbit
Baxter et al [27], 2022	High satisfaction with visual rewards; 75% found the timer motivating	User satisfaction with app’s usability, responsiveness, and animations	NR	Distance measurement for inspiratory detection needed improvement	App required further technical refinement to improve microphone sensitivity
Oberschmidt et al [28], 2022	Exergames promoted challenge and seeing results, motivating participants	NR	NR	Issues with camera accuracy during exercise detection	Technical errors with exercise detection and loud notifications disrupted patient comfort
Finkelstein et al [29], 2023	Positive feedback for visual feedback, ease of navigation, and VR app structure	NR	NR	Simplified controls and interface for older adults with limited computer skills	Minor difficulties in finding and starting the app initially
Gabriel et al [30], 2023	NR	High satisfaction with visual feedback and educational content (mean posttask scores: 4.74-4.89 [out of 5])	NR	Simplified interface and navigation for older adults with limited technological experience	Minor difficulties in initial navigation and setup
Gabriel et al [31], 2023	High engagement; increased focus during exercises; minimal distractions	Improved motivation, focus on exercise content, and engagement	NR	Simplified interface for older adults with limited technological skills	Difficulty with headset weight, loading screens, and initial app navigation
Pancini et al [32], 2023	High engagement anticipated due to immersive VR and personalized savoring exercises	Enhanced positive emotions and psychological well-being	NR	Simplified interface to ensure ease of use for older adult patients	NR
Pardos et al [33], 2023	Scoring system with credits aimed at enhancing patient engagement	NR	NR	Data from smartwatches and Bluetooth devices integrated for monitoring	Further development required to expand recommendation domains
Colombo et al [34], 2024	86.85% attendance rate	High user engagement (mean Short Flow State Scale score 4.40, SD 0.36); fatigue and dyspnea improvements	Focused on older Italian patients with COPD	Use of semi-immersive VR to suit hospital settings	Issues with scaling workload increments
Jin et al [35], 2024	82.5% adherence in the intervention group	NR	Tailored to older Chinese patients with COPD	Visual feedback and game variety catered to balance and respiratory issues	Unclear measurement of exercise intensity
Kizmaz et al [36], 2024	VR+PR^o^ group had significantly longer pedaling time (508.44 s vs 357.56 s; *P*=.007)	NR	NR	Real-world footage of cycling in a forest used to enhance ecological realism	One patient could not continue due to dizziness related to VR use
McAnirlin et al [10], 2024	Participants cocreated their own VR experiences, leading to high engagement and satisfaction	NR	Customized to individual preferences and memories	Personalized VR experiences were created using 360-degree videos	Customization required multiple visits and effort to personalize scenes

^a^CSQ-8: Client Satisfaction Questionnaire-8.

^b^CG: control group.

^c^NR: not reported.

^d^EG: experimental group.

^e^VGS: virtual game systems.

^f^COPD: chronic obstructive pulmonary disease.

^g^GRAIL: Gait Real-time Analysis Interactive Lab.

^h^VR: virtual reality.

^i^ICT: information and communication technology.

^j^AR: augmented reality.

^k^QoL: quality of life.

^l^AVG: active video game.

^m^GEQ: Game Engagement Questionnaire.

^n^IMI: Intrinsic Motivation Inventory.

^o^PR: pulmonary rehabilitation.

### Risk of Bias and Limitations

The reviewed studies presented varying levels of bias and limitations. For the RCTs, additional considerations included randomization procedures and blinding, while the non-RCT studies were evaluated based on their specific study design characteristics. Many of the studies (28/29, 97%), particularly early-stage trials, did not formally assess the risk of bias, often resulting in moderate risk due to factors such as small sample sizes and a lack of long-term follow-up [8,14]. Only a few studies (1/29, 3%), such as the one by Rutkowski et al [9], used formal tools and structured randomization to minimize bias, resulting in a lower risk profile.

Common limitations included a reliance on small sample sizes and subjective data, particularly in studies using self-reported outcomes, which may introduce reporting bias [25]. Technical challenges, such as issues with device accuracy and system usability, also affected study outcomes, as seen in the studies by Tabak et al [8] and Oberschmidt et al [28]. Furthermore, most of the studies (13/29, 45%) lacked long-term follow-up, limiting their ability to evaluate sustained effects of the interventions.

Table 6 provides a detailed breakdown of each study’s risk-of-bias assessment, findings, and limitations.

**Table 6:** Risk of bias and study limitations.

Study	Risk-of-bias assessment	Risk-of-bias findings	Limitations
Tabak et al [8], 2014	NR^a^	Moderate risk (selection and attrition biases)	Small sample, high dropout (86% in CG^b^), and low exercise adherence (21%)
Mazzoleni et al [14], 2014	NR	Moderate risk (small sample size and lack of blinding)	Small sample, short duration, and lack of long-term follow-up
Kotrach et al [15], 2015	NR	Moderate risk (small sample size and preliminary data)	Small sample size, lack of long-term follow-up, and exclusion due to language barriers
Hoaas et al [12], 2016	NR	Moderate risk (small sample size)	Small sample size and seasonal effects on adherence
LeGear et al [16], 2016	NR	Moderate risk (small sample size and short duration)	Small sample size, lack of long-term follow-up, and supervision needed for safe exercise
Liu et al [17], 2016	NR	Moderate risk (lack of randomization and single-site study)	Small sample size, lack of long-term follow-up, monocentric, and limited applicability to patients classified as GOLD^c^ stage 4
Bamidis et al [18], 2017	Not assessed in early stages of project	Risk of bias expected due to self-reported data and social desirability	No long-term results yet; potential for socioeconomic and geographic disparities in outcomes
Burkow et al [19], 2018	NR	Low generalizability due to small sample size and bias from prior rehabilitation experience	Small sample size, self-reported activity data, and lack of a CG
De Las Heras et al [20], 2018	NR	Moderate risk (small sample size and subjective feedback)	Small sample size and lack of long-term follow-up; only Nordic countries involved
Parent et al [21], 2018	NR	Moderate risk (small sample size; single session)	Short study duration, small sample size, and lack of long-term follow-up
Rutkowski et al [22], 2019	NR	Moderate risk (short intervention duration)	Short duration, lack of long-term follow-up, and no blinding of participants
Sutanto et al [23], 2019	NR	Moderate risk (small sample size)	Small sample size, unblinded study, and lack of intensity monitoring for the Wii exercises
Jung et al [13], 2020	NR	Moderate risk (small sample size and acknowledged limitations)	Small sample size and limited generalizability
Rutkowski et al [11], 2020	NR	Low risk (structured randomization and CG)	Short duration (2 wk); only patients classified as GOLD stages 2 and 3 included
Tu et al [24], 2020	NR (demonstration phase)	NR	Small-scale demonstration, short duration, and lack of long-term data
Rutkowski et al [9], 2021	Assessor-blinded RCT^d^ with controlled randomization (low risk of bias)	Low risk (structured randomization and CG)	Short duration; only hospital based
Simmich et al [25], 2021	NR	Low risk (small sample size and self-reported data)	Lack of generalizability due to the small sample size and limited geographic representation
Simmich et al [26], 2021	NR	Possible bias due to the involvement of the EG^e^ in co-design	Small sample size, short trial duration, Fitbit issues, and lack of notifications in the game
Baxter et al [27], 2022	NR	Low risk (randomization and crossover design)	Small sample, short session duration, and lack of clinical participants
Oberschmidt et al [28], 2022	NR	Some dropouts due to exacerbation but not directly related to intervention	Small sample size, dropouts after 12 wk, and occasional technical issues
Finkelstein et al [29], 2023	NR	Low risk (all participants completed the tasks without significant issues)	Small sample size; lack of long-term follow-up
Gabriel et al [30], 2023	NR	Low risk (comprehensive task completion by all participants)	Small sample size, no CG, and lack of long-term follow-up
Gabriel et al [31], 2023	NR	Low risk (most participants completed the tasks easily)	Small sample size, short duration, and lack of long-term follow-up
Pancini et al [32], 2023	NR (planned study)	NR (study pending implementation)	Small sample size; short intervention duration
Pardos et al [33], 2023	NR (pilot)	NR	Exclusion of factors such as nutrition, smoking, and drinking; system still in development
Colombo et al [34], 2024	NR	Low risk (high adherence and positive outcomes)	Small sample size; no CG
Jin et al [35], 2024	NR	Low risk (group similarity and controlled environment)	Lack of long-term adherence tracking; reliance on self-report
Kizmaz et al [36], 2024	NR	Likely low risk, given the blinded evaluator and randomized design	No objective assessment of cybersickness or patient satisfaction; no third group for comparison with usual care
McAnirlin et al [10], 2024	NR	NR	Small sample size, no CG, and exploratory design

^a^NR: not reported.

^b^CG: control group.

^c^GOLD: Global Initiative for Chronic Obstructive Lung Disease.

^d^RCT: randomized controlled trial.

^e^EG: experimental group.

## Discussion

### Synthesis of Findings

#### Effectiveness in COPD Management

The reviewed studies highlighted the positive impact of gamified interventions on physical outcomes, QoL, and patient engagement in COPD management; for instance, interventions incorporating VR or exergames, such as those by Mazzoleni et al [14] and Jung et al [13], showed improved exercise tolerance with significant gains in the 6-minute walk test. Similarly, gamified tools often led to enhanced QoL, as patients reported reduced anxiety and a sense of achievement due to positive reinforcement mechanisms [9,32]. Such outcomes align with broader trends noted in the literature, where eHealth interventions had a positive effect on exercise endurance and QoL in patients with COPD [5]. Through interactive and motivational features, gamified interventions provide both physiological and psychological benefits, which seem crucial for managing COPD symptoms more effectively.

#### Engagement and Adherence

Gamified interventions demonstrated high engagement and adherence due to interactive features such as real-time feedback and immersive environments. Studies using VR or gamified apps, such as the ones by Tu et al [24] and Hoaas et al [12], reported consistently high engagement rates, with patients maintaining adherence due to motivational tools such as rewards and feedback loops [27,29]. These methods seem to support sustained behavioral changes necessary for chronic disease management [19]. Similar findings were echoed in reviews where gamification substantially enhanced adherence in COPD and other chronic disease management contexts [2,6]. Consequently, integrating gamified elements into COPD management is effective in maintaining long-term adherence, critical for preventing exacerbations.

#### Comparative Analysis With Nongamified Interventions

When comparing gamified and nongamified approaches, the evidence suggests that gamification provides superior patient engagement and adherence outcomes; for example, Chang et al [5] found that while telemonitoring improved exercise endurance, VR and game-based interventions fostered higher satisfaction. Liu et al [2] further emphasized that VR-based gamification was particularly beneficial in promoting exercise adherence, especially among patients with COPD who find traditional exercises repetitive. In addition, Selles et al [1] found that gamified components significantly increased physical activity in patients with chronic disease, outperforming conventional approaches. Similarly, Obrero-Gaitán et al [3] noted that VR gamified interventions enhanced motivation and enjoyment, making COPD management more accessible and engaging. These findings underscore that gamified strategies may provide a more effective, patient-centered approach to COPD care.

#### Main Findings in RCT Studies

RCTs are effective in reducing bias; controlling for external variables; and providing more precise and broadly applicable results, which is especially important in intervention studies of complex diseases such as COPD. To highlight the results from higher-quality studies, we further discuss the main findings of the RCTs (11/29, 38%) included in this review. Several of the RCTs (7/11, 64%) reported significant improvements in physical fitness and exercise capacity, including 6-minute walk distance, exercise tolerance, and performance on the Senior Fitness Test. Specifically, experimental groups showed greater improvements in these areas than control groups, indicating enhanced physical endurance and exercise capacity [11,14,22,23,26,35,36]. Another finding is that, compared to control groups, intervention groups demonstrated significant improvements in emotional well-being and mental health indicators, such as anxiety, depression, and stress [9,32]. However, there were also RCTs (1/29, 3%) that found no statistically significant difference in clinical outcomes between the groups [8].

### Strengths and Limitations

#### Strengths

Gamified interventions offer unique strengths in COPD management, particularly in enhancing patient engagement and supporting home-based or self-managed care. By incorporating features such as real-time feedback, interactive environments, and personalized adjustments, gamified tools address some of the limitations of traditional, clinic-based programs [8,13]. These interventions leverage technology to empower patients to monitor their symptoms, engage in rehabilitation activities, and adhere to exercise routines from home, providing significant flexibility and independence, which are highly valued in chronic care [6]. In addition, gamified tools have shown positive psychological effects, with many participants reporting improvements in mood, motivation, and self-efficacy [9,32]. This approach aligns well with the growing shift toward patient-centered care, emphasizing autonomy and self-management in chronic disease contexts such as COPD [3].

#### Limitations

Despite their strengths, the reviewed studies revealed several methodological challenges that limit the generalizability and consistency of the findings. One primary limitation was the heterogeneity across study designs, intervention types, and technology platforms, making it difficult to draw consistent conclusions about intervention efficacy; for example, while some of the studies (15/29, 52%) used VR-based environments, others (14/29, 48%) used simple exergames or mobile apps, leading to varied engagement and adherence outcomes [14,17]. Limited sample sizes and short intervention durations were also frequent issues, with some of the studies (15/29, 52%) having <20 participants or lasting only a few weeks [12,15]. Technical issues such as usability challenges and the need for advanced equipment can create barriers for older adults with limited technological familiarity [1,2]. Furthermore, a formal risk-of-bias assessment using validated scales, such as the Physiotherapy Evidence Database scale, was not conducted in this review; while we critically appraised the included studies based on study design, sample size, and methodological rigor, the absence of a standardized quality-scoring system may limit the comparability of study quality across different methodologies. Finally, while the inclusion of the China National Knowledge Infrastructure database allowed us to capture studies relevant to the Chinese population, it might limit the reproducibility of the work for researchers without access to this database. These factors contribute to biases and limitations in existing literature, indicating a need for larger, longer-term trials to assess gamified interventions effectively.

### Potential of Gamified Interventions in COPD Management

Digital health technologies and gamification hold considerable potential as transformative approaches for managing chronic respiratory diseases such as COPD. By making rehabilitation more interactive and accessible, these interventions can support sustained patient engagement between medical presentations for exacerbations and beyond traditional clinical settings, addressing key challenges in long-term COPD care [5]. The ability of gamified interventions to integrate with real-time monitoring and provide personalized feedback aligns well with the goals of self-managed, home-based COPD care, making it easier for patients to monitor their symptoms and manage exacerbations effectively [24,29]. In addition, gamification encourages continuous engagement, which is crucial for adherence to exercise routines in COPD rehabilitation [6]. As research advances, digital health and gamification could become essential tools in the COPD management toolkit, offering a scalable, patient-centered approach to chronic respiratory care.

### Conclusions

This systematic review highlights the positive impact of gamified interventions on COPD management, demonstrating improvements in physical outcomes, patient engagement, and QoL. Various studies have shown that gamified tools—ranging from exergames to VR-based PR—enhance patients’ adherence to exercise routines and promote self-management [8,9]. In particular, features such as real-time feedback, interactive guidance, and personalized adjustments make these interventions highly engaging and effective, aligning with the chronic, self-managed nature of COPD care. However, the studies also reveal challenges, such as technological barriers and limitations in sample size and duration, suggesting that more robust trials are needed to confirm the efficacy of gamified interventions in COPD management.

The integration of gamified interventions into COPD care presents significant opportunities for patients, health care providers, and policy makers. Gamified interventions empower patients with interactive and flexible tools for managing COPD symptoms, promoting independence and engagement in their care. These tools offer a novel approach to supporting patient adherence to home-based rehabilitation, reducing the reliance on in-clinic visits. Policy makers can leverage this approach to address the rising costs of COPD management by supporting digital health policies that encourage the development and use of gamified COPD interventions. Such policies could also promote accessibility to these tools across various populations, making COPD care more equitable and scalable.

Future research should focus on addressing the identified limitations of many of the studies in this review by conducting larger, long-term RCTs to assess the sustained impact of gamified interventions on COPD outcomes. Developing culturally relevant and adapted gamified tools can enhance the global applicability of these interventions. Collaborating with gaming companies could facilitate the integration of advanced game mechanics and elements, making interventions more engaging and effective. Furthermore, integrating personalized gamification approaches that cater to individual patient needs and preferences into health care could enhance adherence. Finally, exploring the long-term effects of gamified interventions will be essential for establishing their place in the chronic management of COPD and similar conditions.

## Data Availability

All data generated or analyzed during this study are included in this published paper and its supplementary information files.

## Appendix

Multimedia Appendix 1 Detailed search strategies for each database.

Multimedia Appendix 2 PRISMA (Preferred Reporting Items for Systematic Reviews and Meta-Analyses) checklist.

Multimedia Appendix 3 Template for the data extraction and analysis form.

Multimedia Appendix 4 Details of data extraction and analysis of each reviewed study.

## Abbreviations

COPD: chronic obstructive pulmonary disease
PR: pulmonary rehabilitation
PRISMA: Preferred Reporting Items for Systematic Reviews and Meta-Analyses
QoL: quality of life
RCT: randomized controlled trial
VR: virtual reality
