# Supplementary material for: The Impact of Gamified Interventions on the Management of Chronic Obstructive Pulmonary Disease: Systematic Literature Review
Source: JMIR Serious Games. 2025 May 30;13:e69510. doi: 10.2196/69510 (PMC12166322; doi:10.2196/69510)
Supplement: Multimedia Appendix 1 [file games_v13i1e69510_app1.docx]

## **Appendix 1: Detailed search strategies for each database**

| **Databases** | **Query** | **Articles Founded** |
| --- | --- | --- |
| **PubMed** | ("chronic obstruct* pulmon* disease"[All Fields] OR "COPD"[All Fields] OR "pulmon* disease chronic obstruct*"[All Fields] OR "chronic bronchit*"[All Fields] OR "emphysem*"[All Fields] OR "respirator* disease"[All Fields] OR "pulmon* disorder"[All Fields]) AND ("gamif*"[All Fields] OR "Game-based"[All Fields] OR "serious game*"[All Fields] OR "Gaming"[All Fields] OR "digital game*"[All Fields] OR "health game*"[All Fields] OR "game mechanic*"[All Fields] OR "mobile game*"[All Fields] OR "interactive game*"[All Fields] OR "exergame*"[All Fields] OR (("behavior"[MeSH Terms] OR "behavior"[All Fields] OR "behavioral"[All Fields] OR "behavioural"[All Fields] OR "behavior s"[All Fields] OR "behaviorally"[All Fields] OR "behaviour"[All Fields] OR "behaviourally"[All Fields] OR "behaviours"[All Fields] OR "behaviors"[All Fields] OR "pattern"[All Fields] OR "pattern s"[All Fields] OR "patternability"[All Fields] OR "patternable"[All Fields] OR "patterned"[All Fields] OR "patterning"[All Fields] OR "patternings"[All Fields] OR "patterns"[All Fields]) AND "gamif*"[All Fields]) OR "Virtual Reality"[All Fields] OR "Augmented Reality"[All Fields]) AND ("self manage*"[All Fields] OR "Self-Care"[All Fields] OR "disease manage*"[All Fields] OR "chronic disease manage*"[All Fields] OR "symptom monitor*"[All Fields] OR "pulmon* rehab*"[All Fields] OR "pulmon* rehabilitat*"[All Fields] OR "exacerbat* prevent*"[All Fields] OR "patient engage*"[All Fields] OR "patient adher*"[All Fields] OR "treat* adher*"[All Fields] OR "health monitor*"[All Fields] OR "behavior* change"[All Fields] OR "behaviour* change"[All Fields] OR "condition manage*"[All Fields] OR "clinical monitor*"[All Fields] OR "intervent* manage*"[All Fields]) | 44 |
| **Scopus** | ( ALL ( "Chronic Obstruct* Pulmon* Disease" OR "COPD" OR "Pulmon* Disease, Chronic Obstruct*" OR "Chronic Bronchit*" OR "Emphysem*" OR "Respirator* Disease" OR "Pulmon* Disorder" ) AND ALL ( "Gamif*" OR "Game-based" OR "Serious Game*" OR "Gaming" OR "Digital Game*" OR "Health Game*" OR "Game Mechanic*" OR "Mobile Game*" OR "Interactive Game*" OR "Exergame*" OR "Behavioral Gamif*" OR "Virtual Reality" OR "Augmented Reality" ) AND ALL ( "Self-Manage*" OR "Self-Care" OR "Disease Manage*" OR "Chronic Disease Manage*" OR "Symptom Monitor*" OR "Pulmon* Rehab*" OR "Pulmon* Rehabilitat*" OR "Exacerbat* Prevent*" OR "Patient Engage*" OR "Patient Adher*" OR "Treat* Adher*" OR "Health Monitor*" OR "Behavior* Change" OR "Behaviour* Change" OR "Condition Manage*" OR "Clinical Monitor*" OR "Intervent* Manage*" ) ) AND PUBYEAR > 2013 AND PUBYEAR < 2025 AND ( LIMIT-TO ( DOCTYPE , "ar" ) ) AND ( LIMIT-TO ( LANGUAGE , "English" ) ) | 880 |
| **Cochrane Library** | “Chronic Obstruct* Pulmon* Disease” OR “COPD” OR “Pulmon* Disease, Chronic Obstruct*” OR “Chronic Bronchit*” OR “Emphysem*” OR “Respirator* Disease” OR “Pulmon* Disorder” in All Text AND “Gamif*” OR “Game-based” OR “Serious Game*” OR “Gaming” OR “Digital Game*” OR “Health Game*” OR “Game Mechanic*” OR “Mobile Game*” OR “Interactive Game*” OR “Exergame*” OR “Behavioral Gamif*” OR “Virtual Reality” OR “Augmented Reality” in All Text AND “Self-Manage*” OR “Self-Care” OR “Disease Manage*” OR “Chronic Disease Manage*” OR “Symptom Monitor*” OR “Pulmon* Rehab*” OR “Pulmon* Rehabilitat*” OR “Exacerbat* Prevent*” OR “Patient Engage*” OR “Patient Adher*” OR “Treat* Adher*” OR “Health Monitor*” OR “Behavior* Change” OR “Behaviour* Change” OR “Condition Manage*” OR “Clinical Monitor*” OR “Intervent* Manage*” in All Text - (Word variations have been searched) | 12 |
| **Web of Science** | Results for “Chronic Obstruct* Pulmon* Disease” OR “COPD” OR “Pulmon* Disease, Chronic Obstruct*” OR “Chronic Bronchit*” OR “Emphysem*” OR “Respirator* Disease” OR “Pulmon* Disorder” (All Fields) AND “Gamif*” OR “Game-based” OR “Serious Game*” OR “Gaming” OR “Digital Game*” OR “Health Game*” OR “Game Mechanic*” OR “Mobile Game*” OR “Interactive Game*” OR “Exergame*” OR “Behavioral Gamif*” OR “Virtual Reality” OR “Augmented Reality” (All Fields) AND “Self-Manage*” OR “Self-Care” OR “Disease Manage*” OR “Chronic Disease Manage*” OR “Symptom Monitor*” OR “Pulmon* Rehab*” OR “Pulmon* Rehabilitat*” OR “Exacerbat* Prevent*” OR “Patient Engage*” OR “Patient Adher*” OR “Treat* Adher*” OR “Health Monitor*” OR “Behavior* Change” OR “Behaviour* Change” OR “Condition Manage*” OR “Clinical Monitor*” OR “Intervent* Manage*” (All Fields) | 55 |
| **Embase** | ("Chronic Obstruct* Pulmon* Disease" or "COPD" or "Pulmon* Disease, Chronic Obstruct*" or "Chronic Bronchit*" or "Emphysem*" or "Respirator* Disease" or "Pulmon* Disorder").mp. and ("Gamif*" or "Game-based" or "Serious Game*" or "Gaming" or "Digital Game*" or "Health Game*" or "Game Mechanic*" or "Mobile Game*" or "Interactive Game*" or "Exergame*" or "Behavioral Gamif*" or "Virtual Reality" or "Augmented Reality").mp. and ("Self-Manage*" or "Self-Care" or "Disease Manage*" or "Chronic Disease Manage*" or "Symptom Monitor*" or "Pulmon* Rehab*" or "Pulmon* Rehabilitat*" or "Exacerbat* Prevent*" or "Patient Engage*" or "Patient Adher*" or "Treat* Adher*" or "Health Monitor*" or "Behavior* Change" or "Behaviour* Change" or "Condition Manage*" or "Clinical Monitor*" or "Intervent* Manage*").mp. [mp=title, abstract, heading word, drug trade name, original title, device manufacturer, drug manufacturer, device trade name, keyword heading word, floating subheading word, candidate term word] | 65 |
| **IEEExplore** | ("Full Text Only":“Chronic Obstructive Pulmonary Disease” OR "Full Text Only":“COPD” OR "Full Text Only":“Pulmonary Disease, Chronic Obstructive” OR "Full Text Only":“Chronic Bronchit*” OR "Full Text Only":“Emphysem*” OR "Full Text Only":“Respiratory Disease” OR "Full Text Only":“Pulmonary Disorder”) AND ("Full Text Only":“Gamif*” OR "Full Text Only":“Game-based” OR "Full Text Only":“Serious Game” OR "Full Text Only":“Gaming” OR "Full Text Only":“Digital Game” OR "Full Text Only":“Health Game” OR "Full Text Only":“Game Mechanic*” OR "Full Text Only":“Mobile Game” OR "Full Text Only":“Interactive Game” OR "Full Text Only":“Exergame*” OR "Full Text Only":“Behavioral Gamif*” OR "Full Text Only":“Virtual Reality” OR "Full Text Only":“Augmented Reality”) AND ("Full Text Only":“Self-Management” OR "Full Text Only":“Self-Care” OR "Full Text Only":“Disease Management” OR "Full Text Only":“Chronic Disease Management” OR "Full Text Only":“Symptom Monitoring” OR "Full Text Only":“Pulmonary Rehabilitation” OR "Full Text Only":“Pulmonary Rehabilitation” OR "Full Text Only":“Exacerbat* Prevent*” OR "Full Text Only":“Patient Engagement” OR "Full Text Only":“Patient Adherence” OR "Full Text Only":“Treatment Adherence” OR "Full Text Only":“Health Monitoring” OR "Full Text Only":“Behavior Change” OR "Full Text Only":“Behaviour Change” OR "Full Text Only":“Condition Management” OR "Full Text Only":“Clinical Monitor*” OR "Full Text Only":“Intervention Management”)  Filter Applied: 2014-2024; Journals | 518 |
| **CNKI (China National Knowledge Infrastructure)** | (Title, Keyword and Abstract : "Chronic Obstruct* Pulmon* Disease" OR "COPD" OR "Pulmon* Disease, Chronic Obstruct*" OR "Chronic Bronchit"" O R "Emphyse (Fuzzy)) AND (Title, Keyword and Abstract : "Gamif*" OR "Game-based" OR "Serious Game*' OR "Gaming" OR "Digital Game*' OR *H ealth Game*™ OR "Game Mechanic*" OR "Mob (Fuzzy)) AND (Title, Keyword and Abstract : "Self-Manage*" OR "Self-Care" OR "Disease Manage OR "Chronic Disease Manage*" OR "Symptom Monitor*' OR "Pulmon* Rehab* (Fuzzy)) | 0 |
| **Total** |  | 1574 |
