# Supplementary material for: The Impact of Gamified Interventions on the Management of Chronic Obstructive Pulmonary Disease: Systematic Literature Review
Source: JMIR Serious Games. 2025 May 30;13:e69510. doi: 10.2196/69510 (PMC12166322; doi:10.2196/69510)
Supplement: Multimedia Appendix 3 [file games_v13i1e69510_app3.docx]

## **Appendix 3: Template for Data Extraction and Analysis Form**

| **Study No.** | | | |
| --- | --- | --- | --- |
| **Section** | **Q#** | **Data Extracted** | **Details** |
| **Study Information** | 1 | Study ID | Unique identifier for the study (e.g., author, year) |
|  | 2 | Title | Full title of the study |
|  | 3 | Authors | Names of the authors |
|  | 4 | Year of Publication | Year when the study was published |
|  | 5 | Journal/Source | Name of the journal or source where the study was published |
| **Study Design** | 6 | Study Type | RCT, observational study, cohort, case study, etc. |
|  | 7 | Sample Size | Number of participants in the study |
|  | 8 | Study Setting | Location and setting of the study (e.g., hospital, home, clinic) |
|  | 9 | Intervention Duration | Length of time the intervention was applied |
| **Intervention Characteristics** | 10 | Type of Gamified Intervention | Description of the gamification used (e.g., serious game, exergame) |
|  | 11 | Platform/Technology Used | Smartphone app, VR, AR, wearables, etc. |
|  | 12 | Game Elements | Points, rewards, challenges, levels, leaderboards, etc. |
|  | 13 | Tailoring/Personalization | Adaptation to individual patient’s needs (if applicable) |
|  | 14 | Integration with Healthcare | Integration with healthcare systems, monitoring, feedback |
| **Participant Characteristics** | 15 | Population | Description of participants (age, gender, disease severity, etc.) |
|  | 16 | Inclusion/Exclusion Criteria | Key criteria for participant selection |
|  | 17 | Baseline Characteristics | Relevant baseline data (e.g., lung function, exacerbation rates) |
| **Outcomes** | 18 | Primary Outcomes | Lung function, exacerbation rates, quality of life, etc. |
|  | 19 | Secondary Outcomes | Adherence, engagement, user satisfaction, behavioral change |
|  | 20 | Effectiveness Results | Summary of main results regarding effectiveness |
|  | 21 | Behavioral Outcomes | Any reported impact on behavior, motivation, or self-management |
|  | 22 | Engagement Metrics | User participation, retention, and engagement levels |
|  | 23 | Quality of Life Improvements | Impact on quality of life (e.g., SGRQ, SF-36 scores) |
| **Study Quality and Bias** | 24 | Risk of Bias Assessment | Tool used (e.g., Cochrane Risk of Bias, Newcastle-Ottawa scale) |
|  | 25 | Risk of Bias Findings | High, medium, or low risk of bias |
|  | 26 | Limitations | Limitations reported by the authors or identified during extraction |
| **Adaptation** | 27 | Cultural or Demographic Adaptation | Any cultural or demographic adjustments made to the gamified intervention |
|  | 28 | Technical Adaptation | Adaptation to different devices, platforms, or accessibility needs |
|  | 29 | Challenges in Adaptation | Any reported challenges in implementing or adapting the intervention |
| **Conclusions** | 30 | Study Conclusion | Main conclusion drawn by the authors |
|  | 31 | Relevance to Gamified COPD Management | How relevant the study is to gamified COPD management |
|  | 32 | Recommendations for Future Research | Suggestions for future studies or areas to be explored |
