# Supplementary material for: The Impact of Gamified Interventions on the Management of Chronic Obstructive Pulmonary Disease: Systematic Literature Review
Source: JMIR Serious Games. 2025 May 30;13:e69510. doi: 10.2196/69510 (PMC12166322; doi:10.2196/69510)
Supplement: Multimedia Appendix 4 [file games_v13i1e69510_app4.docx]

## Appendix 4: Details of Data Extraction and Analysis of Each Reviewed Study

#### Appendix 3-1: Tabak et al., 2014

| **Study 1** | | | |
| --- | --- | --- | --- |
| **Section** | **Q#** | **Data Extracted** | **Details** |
| **Study Information** | 1 | Study ID | Tabak et al., 2014 |
|  | 2 | Title | A telehealth program for self-management of COPD exacerbations and promotion of an active lifestyle: A pilot randomized controlled trial |
|  | 3 | Authors | Monique Tabak, Marjolein Brusse-Keizer, Paul van der Valk, Hermie Hermens, Miriam Vollenbroek-Hutten |
|  | 4 | Year of Publication | 2014 |
|  | 5 | Journal/Source | *International Journal of COPD* |
| **Study Design** | 6 | Study Type | RCT |
|  | 7 | Sample Size | N=29 |
|  | 8 | Study Setting | Hospital and primary care physiotherapy practices, Enschede, Netherlands |
|  | 9 | Intervention Duration | 9 months |
| **Intervention Characteristics** | 10 | Type of Gamified Intervention | Motivational cues |
|  | 11 | Platform/Technology Used | Wearable device, smartphone app, web-based portal |
|  | 12 | Game Elements | Motivational cues, daily feedback |
|  | 13 | Tailoring/Personalization | Exercise schemes tailored by physiotherapists |
|  | 14 | Integration with Healthcare | Integrated with primary and secondary care, healthcare professionals monitored progress |
| **Participant Characteristics** | 15 | Population | Adults with COPD (mean age 64) |
|  | 16 | Inclusion/Exclusion Criteria | Regular exacerbations, computer with internet required |
|  | 17 | Baseline Characteristics | FEV1 % predicted: Telehealth 50.0%, Control 36.0%; Exercise capacity (6MWT): Telehealth 409.5m, Control 313.0m |
| **Outcomes** | 18 | Primary Outcomes | Exacerbations: EG 33 (median 2.0);  Hospitalizations: EG 4 (median 5.5 days), CG 5 (median 7.0 days);  Quality of life (EQ-5D VAS): EG 72.3, CG 62.4 |
|  | 19 | Secondary Outcomes | Adherence to web portal: 86.4% of days; Adherence to exercise: 21%; Satisfaction (CSQ-8): EG 26.4/32, CG 30.4/32 |
|  | 20 | Effectiveness Results | Exacerbations: 33 in EG; Exercise adherence: 21%; Satisfaction: EG 26.4/32; No statistically significant difference in clinical outcomes between groups |
|  | 21 | Behavioral Outcomes | Improved self-management of exacerbations (86.4% diary adherence) |
|  | 22 | Engagement Metrics | Web portal usage: 86.4% of days; Exercise adherence: 21%; Activity coach used for 299 days (132 days monitoring, 167 days feedback) |
|  | 23 | Quality of Life Improvements | EQ-5D VAS score: EG 64.7 (baseline) to 72.3 (3 months); CG 65.0 (baseline) to 62.4 (3 months);  CCQ score: EG 2.0 (baseline) to 1.8 (3 months) |
| **Study Quality and Bias** | 24 | Risk of Bias Assessment | NA |
|  | 25 | Risk of Bias Findings | Moderate risk (selection and attrition biases) |
|  | 26 | Limitations | Small sample, high dropout (86% in control), low exercise adherence (21%) |
| **Adaptation** | 27 | Cultural or Demographic Adaptation | NA |
|  | 28 | Technical Adaptation | Wearable and web-based portal; no adaptation for other platforms |
|  | 29 | Challenges in Adaptation | Technical issues with activity coach (e.g., cycling accuracy), low exercise adherence (21%) |
| **Conclusions** | 30 | Study Conclusion | High satisfaction but low adherence to exercise; professionals crucial for adherence |
|  | 31 | Relevance to Gamified COPD Management | Moderate, as motivational cues align with gamified principles |
|  | 32 | Recommendations for Future Research | Full-scale implementation, gamification to improve engagement, cost-effectiveness studies |

#### Appendix 3-2: Mazzoleni et al., 2014

| **Study 2** | | | |
| --- | --- | --- | --- |
| **Section** | **Q#** | **Data Extracted** | **Details** |
| **Study Information** | 1 | Study ID | Mazzoleni et al., 2014 |
|  | 2 | Title | Interactive videogame as rehabilitation tool of patients with chronic respiratory diseases: Preliminary results of a feasibility study |
|  | 3 | Authors | Stefano Mazzoleni, Giulia Montagnani, Guido Vagheggini, Lorenzo Buono, Francesca Moretti, Paolo Dario, Nicolino Ambrosino |
|  | 4 | Year of Publication | 2014 |
|  | 5 | Journal/Source | *Respiratory Medicine* |
| **Study Design** | 6 | Study Type | RCT |
|  | 7 | Sample Size | N=40 (20 EG, 20 CG) |
|  | 8 | Study Setting | Auxilium Vitae Rehabilitation Center, Volterra, Italy |
|  | 9 | Intervention Duration | 3 weeks |
| **Intervention Characteristics** | 10 | Type of Gamified Intervention | Exergames using Nintendo Wii Fit Plus |
|  | 11 | Platform/Technology Used | Nintendo Wii, Wii Balance Board |
|  | 12 | Game Elements | Real-time feedback, visual and auditory cues |
|  | 13 | Tailoring/Personalization | Exercise intensity adjusted by physiotherapist |
|  | 14 | Integration with Healthcare | Supervised by healthcare professionals (physicians, nurses, physiotherapists) |
| **Participant Characteristics** | 15 | Population | Mean age: EG = 68.9, CG = 73.5; Chronic respiratory diseases (COPD, ILD, etc.) |
|  | 16 | Inclusion/Exclusion Criteria | Included stable chronic respiratory disease patients; excluded motor limitations, severe conditions |
|  | 17 | Baseline Characteristics | FEV1 % predicted: EG = 66.3%, CG = 59.7%; 6MWT: EG = 253.1m, CG = 230.4m |
| **Outcomes** | 18 | Primary Outcomes | 6MWT: EG +97.4m, CG +61.1m;  TDI score: EG = 3.9, CG = 2.2;  SGRQ: EG -10.8, CG -12.7 |
|  | 19 | Secondary Outcomes | Adherence: All participants completed intervention;  Satisfaction: EG = 42.4, CG = 43.9 |
|  | 20 | Effectiveness Results | Significant improvement in 6MWT and dyspnea for EG compared to CG |
|  | 21 | Behavioral Outcomes | Improved patient motivation and engagement in EG |
|  | 22 | Engagement Metrics | 7 additional Wii Fit sessions for EG, all completed |
|  | 23 | Quality of Life Improvements | SGRQ improved: EG -10.8 points, CG -12.7 points |
| **Study Quality and Bias** | 24 | Risk of Bias Assessment | NA |
|  | 25 | Risk of Bias Findings | Moderate risk (small sample size, lack of blinding) |
|  | 26 | Limitations | Small sample, short duration, no long-term follow-up |
| **Adaptation** | 27 | Cultural or Demographic Adaptation | NA |
|  | 28 | Technical Adaptation | None beyond Wii Fit system |
|  | 29 | Challenges in Adaptation | Initial difficulty with balance board, exclusion of patients with motor limitations |
| **Conclusions** | 30 | Study Conclusion | Interactive videogames are feasible for pulmonary rehab, improving exercise tolerance and dyspnea |
|  | 31 | Relevance to Gamified COPD Management | Moderate, with engagement and real-time feedback relevant to gamified interventions |
|  | 32 | Recommendations for Future Research | Explore long-term effects, gamified elements, and personalization for broader populations |

#### Appendix 3-3: Kotrach et al., 2015

| **Study 3** | | | |
| --- | --- | --- | --- |
| **Section** | **Q#** | **Data Extracted** | **Details** |
| **Study Information** | 1 | Study ID | Kotrach et al., 2015 |
|  | 2 | Title | A pilot study using virtual game system to maintain adherence to home-based exercise following pulmonary rehabilitation in chronic obstructive pulmonary disease |
|  | 3 | Authors | Houssam Kotrach, Esther Dajczman, Geneviève Tremblay, Marcel Baltzan, et al. |
|  | 4 | Year of Publication | 2015 |
|  | 5 | Journal/Source | *Chest* |
| **Study Design** | 6 | Study Type | Pilot RCT |
|  | 7 | Sample Size | N=12 (6 EG, 6 CG) |
|  | 8 | Study Setting | Mount-Sinai Hospital, Montreal, Canada |
|  | 9 | Intervention Duration | Participants received 3 to 6 hours of individualized training, with follow-up at home |
| **Intervention Characteristics** | 10 | Type of Gamified Intervention | Exergames using Nintendo Wii Fit U, focusing on upper and lower extremity exercises |
|  | 11 | Platform/Technology Used | Nintendo Wii Fit U |
|  | 12 | Game Elements | 11 pre-validated games, real-time feedback on performance |
|  | 13 | Tailoring/Personalization | Individualized training sessions based on patient needs |
|  | 14 | Integration with Healthcare | Monitored by physiotherapists during in-hospital and home training |
| **Participant Characteristics** | 15 | Population | Patients with severe to very severe COPD; mean age 64±10 years; mean FEV1 of 0.78±0.25 L |
|  | 16 | Inclusion/Exclusion Criteria | Severe COPD, patients admitted to PR; excluded those with language barriers and inability to use VGS |
|  | 17 | Baseline Characteristics | 6MWD at baseline was 306±81 meters |
| **Outcomes** | 18 | Primary Outcomes | Exercise tolerance (6MWD), heart rate, and oxygen saturation (SpO2) monitored; 6MWD was 306±81 meters at baseline |
|  | 19 | Secondary Outcomes | Heart rate increased from 83±14 bpm to 103±17 bpm;  SpO2 decreased from 95±1.8% to 91±2.4%;  dyspnea increased from 0.5±0.8 to 2.6±0.8 on the Borg scale |
|  | 20 | Effectiveness Results | Preliminary results showed participants could maintain exercise training post-PR using VGS |
|  | 21 | Behavioral Outcomes | Dyspnea and leg discomfort increased, indicating exertion during exercise |
|  | 22 | Engagement Metrics | All participants adhered to the VGS training |
|  | 23 | Quality of Life Improvements | NA |
| **Study Quality and Bias** | 24 | Risk of Bias Assessment | NA |
|  | 25 | Risk of Bias Findings | Moderate risk (small sample size, preliminary data) |
|  | 26 | Limitations | Small sample size, no long-term follow-up, exclusion due to language barriers |
| **Adaptation** | 27 | Cultural or Demographic Adaptation | NA |
|  | 28 | Technical Adaptation | None beyond training sessions |
|  | 29 | Challenges in Adaptation | Language barriers and patient ability to use VGS |
| **Conclusions** | 30 | Study Conclusion | VGS is feasible for maintaining exercise adherence post-PR in COPD patients, but long-term benefits need further evaluation |
|  | 31 | Relevance to Gamified COPD Management | High, given the use of exergames to promote physical activity post-rehabilitation |
|  | 32 | Recommendations for Future Research | Larger studies with long-term follow-up to assess adherence and health outcomes |

#### Appendix 3-4: Hoaas et al., 2016

| **Study 4** | | | |
| --- | --- | --- | --- |
| **Section** | **Q#** | **Data Extracted** | **Details** |
| **Study Information** | 1 | Study ID | Hoaas et al., 2016 |
|  | 2 | Title | Adherence and factors affecting satisfaction in long-term telerehabilitation for patients with chronic obstructive pulmonary disease: a mixed methods study |
|  | 3 | Authors | Hanne Hoaas, Hege Kristin Andreassen, Linda Aarøen Lien, Audhild Hjalmarsen, Paolo Zanaboni |
|  | 4 | Year of Publication | 2016 |
|  | 5 | Journal/Source | *BMC Medical Informatics and Decision Making* |
| **Study Design** | 6 | Study Type | Mixed methods, 2-year pilot study |
|  | 7 | Sample Size | N=10 |
|  | 8 | Study Setting | Norway, home-based with telemonitoring |
|  | 9 | Intervention Duration | 2 years |
| **Intervention Characteristics** | 10 | Type of Gamified Intervention | Focus on telerehabilitation via self-management and telemonitoring |
|  | 11 | Platform/Technology Used | Treadmill, pulse oximeter, iPad with a webpage for telemonitoring |
|  | 12 | Game Elements | Regular feedback and remote support from physiotherapists |
|  | 13 | Tailoring/Personalization | Individually tailored exercise program with telephysiotherapist supervision |
|  | 14 | Integration with Healthcare | Integrated through weekly videoconferencing with telephysiotherapists |
| **Participant Characteristics** | 15 | Population | 55.2 years average age; moderate to severe COPD |
|  | 16 | Inclusion/Exclusion Criteria | Included stable COPD patients, excluded those unable to exercise at home |
|  | 17 | Baseline Characteristics | Varied physical capability; average travel distance to hospital 99 km |
| **Outcomes** | 18 | Primary Outcomes | Average adherence: 43.3% for daily diary, 56.2% for exercise training |
|  | 19 | Secondary Outcomes | Increased self-efficacy, emotional safety, experienced health benefits |
|  | 20 | Effectiveness Results | No dropouts; long-term adherence despite motivational challenges |
|  | 21 | Behavioral Outcomes | Participants reported better self-management and coping with COPD |
|  | 22 | Engagement Metrics | On average, 3 diary entries/week, 1.7 training sessions/week |
|  | 23 | Quality of Life Improvements | Reported improved health, increased capacity for daily activities |
| **Study Quality and Bias** | 24 | Risk of Bias Assessment | NA |
|  | 25 | Risk of Bias Findings | Moderate; small sample size |
|  | 26 | Limitations | Small sample size, seasonal effects on adherence |
| **Adaptation** | 27 | Cultural or Demographic Adaptation | NA |
|  | 28 | Technical Adaptation | iPad and treadmill used to adapt exercise training to home settings |
|  | 29 | Challenges in Adaptation | Some technical difficulties with videoconferencing |
| **Conclusions** | 30 | Study Conclusion | Telerehabilitation feasible for long-term exercise adherence in COPD |
|  | 31 | Relevance to Gamified COPD Management | Moderate; insights into maintaining long-term exercise adherence relevant for gamified interventions |
|  | 32 | Recommendations for Future Research | Focus on motivational strategies, explore peer-support or gaming elements |

#### Appendix 3-5: LeGear et al., 2016

| **Study 5** | | | |
| --- | --- | --- | --- |
| **Section** | **Q#** | **Data Extracted** | **Details** |
| **Study Information** | 1 | Study ID | LeGear et al., 2016 |
|  | 2 | Title | Does a Nintendo Wii exercise program provide similar exercise demands as a traditional pulmonary rehabilitation program in adults with COPD |
|  | 3 | Authors | Tyler LeGear, Mark LeGear, Dejan Preradovic, Geoffrey Wilson, Ashley Kirkham, Pat G. Camp |
|  | 4 | Year of Publication | 2016 |
|  | 5 | Journal/Source | *Clinical Respiratory Journal* |
| **Study Design** | 6 | Study Type | Randomized, within-subject cross-over trial |
|  | 7 | Sample Size | N=10 |
|  | 8 | Study Setting | St. Paul’s Hospital, Vancouver, Canada |
|  | 9 | Intervention Duration | Single session with two 15-minute exercise interventions |
| **Intervention Characteristics** | 10 | Type of Gamified Intervention | Exergame using EA Sports Active on Nintendo Wii |
|  | 11 | Platform/Technology Used | Nintendo Wii with SenseWear Armband to track energy expenditure |
|  | 12 | Game Elements | Physical activities including marching, dancing, and punching |
|  | 13 | Tailoring/Personalization | Exercise intensity adjusted based on perceived exertion (3-5 on Borg scale) |
|  | 14 | Integration with Healthcare | Not integrated with broader healthcare; monitored in clinical setting |
| **Participant Characteristics** | 15 | Population | 10 adults (5 male, 5 female) with mean age of 65 years and mean FEV1 58.1% predicted |
|  | 16 | Inclusion/Exclusion Criteria | Included COPD patients completing pulmonary rehab; excluded those with other lung disorders |
|  | 17 | Baseline Characteristics | Mean 6MWT distance: 306±81 meters;  FEV1: 58.1% predicted |
| **Outcomes** | 18 | Primary Outcomes | Energy expenditure: Wii (353.5 J) vs treadmill (317.1 J); Heart rate: Wii (112.5 bpm) vs treadmill (112.7 bpm) |
|  | 19 | Secondary Outcomes | SpO2: Wii (94.7%) vs treadmill (92.3%); RPE: Wii (3.2) vs treadmill (3.1) |
|  | 20 | Effectiveness Results | No significant difference in energy expenditure, heart rate, or perceived exertion between Wii and treadmill |
|  | 21 | Behavioral Outcomes | Participants reported enjoyment and perceived feasibility of Wii exercises at home |
|  | 22 | Engagement Metrics | 90% enjoyed Wii intervention, 80% agreed it could be used at home |
|  | 23 | Quality of Life Improvements | Not specifically reported in this study |
| **Study Quality and Bias** | 24 | Risk of Bias Assessment | NA |
|  | 25 | Risk of Bias Findings | Moderate due to small sample size and short duration |
|  | 26 | Limitations | Small sample size, lack of long-term follow-up, supervision needed for safe exercise |
| **Adaptation** | 27 | Cultural or Demographic Adaptation | NA |
|  | 28 | Technical Adaptation | None beyond standard Wii setup |
|  | 29 | Challenges in Adaptation | Some participants required supervision for safe use |
| **Conclusions** | 30 | Study Conclusion | Wii-based exercises provide similar energy demands as treadmill, suggesting feasibility for COPD rehabilitation |
|  | 31 | Relevance to Gamified COPD Management | Highly relevant, given the use of exergames to maintain exercise engagement in COPD |
|  | 32 | Recommendations for Future Research | Larger, long-term studies needed to assess feasibility, safety, and adherence to home programs |

#### Appendix 3-6: Liu et al., 2016

| **Study 6** | | | |
| --- | --- | --- | --- |
| **Section** | **Q#** | **Data Extracted** | **Details** |
| **Study Information** | 1 | Study ID | Liu et al., 2016 |
|  | 2 | Title | Reproducibility and validity of the 6-minute walk test using the gait real-time analysis interactive lab in patients with COPD and healthy elderly |
|  | 3 | Authors | Wai-Yan Liu, Kenneth Meijer, Jeannet M. Delbressine, Paul J. Willems, Frits M.E. Franssen, Emiel F.M. Wouters, Martijn A. Spruit |
|  | 4 | Year of Publication | 2016 |
|  | 5 | Journal/Source | *PLoS ONE* |
| **Study Design** | 6 | Study Type | Cross-sectional observational study |
|  | 7 | Sample Size | N=61 |
|  | 8 | Study Setting | CIRO, Horn, the Netherlands |
|  | 9 | Intervention Duration | Not applicable (cross-sectional study) |
| **Intervention Characteristics** | 10 | Type of Gamified Intervention | Focus on GRAIL (Gait Real-time Analysis Interactive Lab) technology and 6MWT |
|  | 11 | Platform/Technology Used | GRAIL, 3D motion capture, virtual reality treadmill |
|  | 12 | Game Elements | Virtual reality environment used to simulate walking conditions |
|  | 13 | Tailoring/Personalization | Not personalized beyond adjusting walking pace |
|  | 14 | Integration with Healthcare | No direct integration, though relevant for rehab evaluation |
| **Participant Characteristics** | 15 | Population | COPD patients: mean age 61.9±6.8 years, FEV1/FVC 41.3%; healthy elderly: mean age 61.6±6.1 years, FEV1/FVC 77.1% |
|  | 16 | Inclusion/Exclusion Criteria | COPD patients without neuromuscular or orthopedic conditions; healthy elderly without respiratory or cardiac diseases |
|  | 17 | Baseline Characteristics | COPD patients had 57.6% FEV1 predicted; healthy elderly had 119.1% FEV1 predicted |
| **Outcomes** | 18 | Primary Outcomes | COPD patients walked 27.5m less on GRAIL vs. overground 6MWT; healthy elderly walked 23.6m more on GRAIL |
|  | 19 | Secondary Outcomes | COPD patients: Borg dyspnea 5.4, SpO2 decreased by 7.1%, heart rate increased by 29.5 bpm during overground test |
|  | 20 | Effectiveness Results | GRAIL showed good reproducibility for both groups: ICC of 0.80 for COPD, 0.65 for healthy elderly |
|  | 21 | Behavioral Outcomes | Improved reproducibility and patient engagement with virtual environment for COPD patients |
|  | 22 | Engagement Metrics | 75% of COPD patients and 90% of healthy elderly improved in second GRAIL test |
|  | 23 | Quality of Life Improvements | NA |
| **Study Quality and Bias** | 24 | Risk of Bias Assessment | NA |
|  | 25 | Risk of Bias Findings | Moderate risk due to lack of randomization and single-site study |
|  | 26 | Limitations | Small sample size, no long-term follow-up, monocentric, and limited applicability to GOLD stage IV patients |
| **Adaptation** | 27 | Cultural or Demographic Adaptation | NA |
|  | 28 | Technical Adaptation | No major technical adaptation beyond GRAIL virtual reality setup |
|  | 29 | Challenges in Adaptation | Complex setup required; difficulty for patients using self-paced treadmill |
| **Conclusions** | 30 | Study Conclusion | GRAIL is valid for assessing 6MWD in COPD and healthy elderly; overground 6MWD higher for COPD, lower for healthy elderly on GRAIL |
|  | 31 | Relevance to Gamified COPD Management | Relevant in terms of engaging virtual environments, but no gamification applied |
|  | 32 | Recommendations for Future Research | Need for larger, multi-center studies; new reference values for GRAIL needed for broader applicability |

#### Appendix 3-7: Bamidis et al., 2017

| **Study 7** | | | |
| --- | --- | --- | --- |
| **Section** | **Q#** | **Data Extracted** | **Details** |
| **Study Information** | 1 | Study ID | Bamidis et al., 2017 |
|  | 2 | Title | Multimodal e-Health Services for Smoking Cessation and Public Health: The SmokeFreeBrain Project Approach |
|  | 3 | Authors | Panagiotis D. Bamidis, Evangelos Paraskevopoulos, Evdokimos Konstantinidis, Dimitris Spachos, Antonis Billis |
|  | 4 | Year of Publication | 2017 |
|  | 5 | Journal/Source | *Studies in Health Technology and Informatics* |
| **Study Design** | 6 | Study Type | Multi-level intervention project |
|  | 7 | Sample Size | NA (due to ongoing trial) |
|  | 8 | Study Setting | EU-funded multi-site study |
|  | 9 | Intervention Duration | 3 years |
| **Intervention Characteristics** | 10 | Type of Gamified Intervention | Smoking cessation intervention using gamified apps, mini-games, and social media |
|  | 11 | Platform/Technology Used | Gamification app, neurofeedback, social media, mobile SMS, and pharmacological interventions |
|  | 12 | Game Elements | Tasks, goals, achievements (e.g., “Crushing cigarettes” game, breathing control, exercise gamification) |
|  | 13 | Tailoring/Personalization | Interventions customized by demographic and geographic needs |
|  | 14 | Integration with Healthcare | Public health campaigns, electronic health records (EHR), open data integration |
| **Participant Characteristics** | 15 | Population | Smokers from vulnerable groups: unemployed, COPD, asthma patients |
|  | 16 | Inclusion/Exclusion Criteria | High-risk smokers in High-Middle and Low-Middle Income Countries |
|  | 17 | Baseline Characteristics | Demographic factors included, but no specific baseline values were provided |
| **Outcomes** | 18 | Primary Outcomes | Efficacy of public service announcements (PSAs), e-cigarette interventions, and neurofeedback protocols |
|  | 19 | Secondary Outcomes | Effectiveness in reducing smoking prevalence and improving public health awareness |
|  | 20 | Effectiveness Results | Expected positive impacts on reducing smoking among high-risk groups |
|  | 21 | Behavioral Outcomes | Increased adherence to smoking cessation interventions using gamification and ICT |
|  | 22 | Engagement Metrics | Various engagement tools: achievements, self-reported progress via mobile apps |
|  | 23 | Quality of Life Improvements | Expected improvements in smoking-related morbidity and mortality rates |
| **Study Quality and Bias** | 24 | Risk of Bias Assessment | NA |
|  | 25 | Risk of Bias Findings | Risk of bias expected due to self-reported data and social desirability |
|  | 26 | Limitations | No long-term results yet, potential for socioeconomic/geographical disparities in outcomes |
| **Adaptation** | 27 | Cultural or Demographic Adaptation | Tailored to socioeconomic and cultural contexts of various countries |
|  | 28 | Technical Adaptation | Use of ICT, mobile apps, SMS, and gamification across different platforms |
|  | 29 | Challenges in Adaptation | Interoperability and customization for different healthcare systems, potential digital divide |
| **Conclusions** | 30 | Study Conclusion | SmokeFreeBrain interventions expected to provide scalable and cost-effective smoking cessation strategies |
|  | 31 | Relevance to Gamified COPD Management | High, given focus on COPD patients and use of gamified smoking cessation approaches |
|  | 32 | Recommendations for Future Research | Larger trials to evaluate effectiveness, public health outcomes, and technological integration |

#### Appendix 3-8: Burkow et al., 2018

| **Study 8** | | | |
| --- | --- | --- | --- |
| **Section** | **Q#** | **Data Extracted** | **Details** |
| **Study Information** | 1 | Study ID | Burkow et al., 2018 |
|  | 2 | Title | Promoting exercise training and physical activity in daily life: A feasibility study of a virtual group intervention for behaviour change in COPD |
|  | 3 | Authors | Tatjana M. Burkow, Lars K. Vognild, Elin Johnsen, Astrid Bratvold, Marijke J. Risberg |
|  | 4 | Year of Publication | 2018 |
|  | 5 | Journal/Source | *BMC Medical Informatics and Decision Making* |
| **Study Design** | 6 | Study Type | Feasibility study |
|  | 7 | Sample Size | N=10 |
|  | 8 | Study Setting | Virtual group-based, home setting |
|  | 9 | Intervention Duration | 6 weeks |
| **Intervention Characteristics** | 10 | Type of Gamified Intervention | Virtual group exercise with follow-along videos and exercise diaries |
|  | 11 | Platform/Technology Used | Tablet-based app |
|  | 12 | Game Elements | Virtual group status, rewards (duckling icons for completed exercises), exercise diary |
|  | 13 | Tailoring/Personalization | Self-chosen individual exercises, including flexibility in indoor/outdoor activities |
|  | 14 | Integration with Healthcare | Peer group exercises combined with remote support from rehabilitation professionals |
| **Participant Characteristics** | 15 | Population | COPD patients (aged 47–74) in GOLD stages I–III |
|  | 16 | Inclusion/Exclusion Criteria | COPD diagnosis, participation in pulmonary rehabilitation, ability to use a touchscreen |
|  | 17 | Baseline Characteristics | 7 females, 3 males; baseline physical activity: average 2.9 sessions/week |
| **Outcomes** | 18 | Primary Outcomes | Increase in physical activity from 2.9 to 5.9 sessions per week during the program |
|  | 19 | Secondary Outcomes | High acceptance, improved adherence to exercise routines, group motivation |
|  | 20 | Effectiveness Results | 77% adherence to group exercises, significant increase in physical activity |
|  | 21 | Behavioral Outcomes | Positive impact on motivation to engage in physical activity |
|  | 22 | Engagement Metrics | Peer monitoring and virtual group updates drove engagement |
|  | 23 | Quality of Life Improvements | Improved well-being and mood reported |
| **Study Quality and Bias** | 24 | Risk of Bias Assessment | NA |
|  | 25 | Risk of Bias Findings | Low generalizability due to small sample size and bias from prior rehabilitation experience |
|  | 26 | Limitations | Small sample size, self-reported activity data, no control group |
| **Adaptation** | 27 | Cultural or Demographic Adaptation | NA |
|  | 28 | Technical Adaptation | Tablet optimized with all other apps disabled |
|  | 29 | Challenges in Adaptation | Minor technical issues (weather widget, activity sensor) |
| **Conclusions** | 30 | Study Conclusion | Tablet-based intervention is feasible and motivates increased physical activity in COPD patients |
|  | 31 | Relevance to Gamified COPD Management | High relevance due to group-based virtual gamification and behavior change techniques |
|  | 32 | Recommendations for Future Research | Larger trials needed with a randomized controlled design and long-term follow-up |

#### Appendix 3-9: De Las Heras et al., 2018

| **Study 9** | | | |
| --- | --- | --- | --- |
| **Section** | **Q#** | **Data Extracted** | **Details** |
| **Study Information** | 1 | Study ID | De Las Heras et al., 2018 |
|  | 2 | Title | Augmented reality glasses as a new tele-rehabilitation tool for home use: Patients’ perception and expectations |
|  | 3 | Authors | J. Cerdán de las Heras, M. Tulppo, A. M. Kiviniemi, O. Hilberg, A. Løkke, S. Ekholm, D. Catalán-Matamoros, E. Bendstrup |
|  | 4 | Year of Publication | 2018 |
|  | 5 | Journal/Source | *European Respiratory Journal Conference, ERS* |
| **Study Design** | 6 | Study Type | Qualitative study with focus groups and semi-structured interviews |
|  | 7 | Sample Size | N=13 |
|  | 8 | Study Setting | Aarhus University Hospital (Denmark) and Oulu University Hospital (Finland) |
|  | 9 | Intervention Duration | NA |
| **Intervention Characteristics** | 10 | Type of Gamified Intervention | Use of AR glasses |
|  | 11 | Platform/Technology Used | Laster WAVƎ augmented reality glasses |
|  | 12 | Game Elements | Visual and audio guidance during exercises |
|  | 13 | Tailoring/Personalization | Adjustable brightness, head fixation, interface improvements based on patient feedback |
|  | 14 | Integration with Healthcare | Remote communication and feedback during telerehabilitation |
| **Participant Characteristics** | 15 | Population | 13 patients (7 COPD, 2 IPF, 4 MI), aged 56–75 years |
|  | 16 | Inclusion/Exclusion Criteria | COPD, IPF, or MI patients; exclusion criteria based on cognitive or physical limitations |
|  | 17 | Baseline Characteristics | Mean age 63.3 years; most participants were male (11/13) |
| **Outcomes** | 18 | Primary Outcomes | Positive perception of AR glasses, particularly ease of use and exercise guidance |
|  | 19 | Secondary Outcomes | Suggestions for improvement: adjustable screen, brightness, head fixation |
|  | 20 | Effectiveness Results | Patients saw value in ARG for telerehabilitation, though some found the design heavy |
|  | 21 | Behavioral Outcomes | Motivation to use ARG for physical exercise and rehabilitation |
|  | 22 | Engagement Metrics | High engagement; 12 out of 13 patients appreciated the AR glasses |
|  | 23 | Quality of Life Improvements | NA |
| **Study Quality and Bias** | 24 | Risk of Bias Assessment | NA |
|  | 25 | Risk of Bias Findings | Moderate bias due to small sample size and subjective feedback |
|  | 26 | Limitations | Small sample size, lack of long-term follow-up, only Nordic countries involved |
| **Adaptation** | 27 | Cultural or Demographic Adaptation | NA |
|  | 28 | Technical Adaptation | Adjustments to glasses design and usability proposed by patients |
|  | 29 | Challenges in Adaptation | Issues with head fixation during movement, brightness control |
| **Conclusions** | 30 | Study Conclusion | Patients found value in ARG for telerehabilitation, but improvements are needed for usability |
|  | 31 | Relevance to Gamified COPD Management | Moderate relevance; insights on AR use in COPD management can inform future gamified tools |
|  | 32 | Recommendations for Future Research | Larger studies needed; technical improvements should focus on usability, personalization |

#### Appendix 3-10: Parent et al., 2018

| **Study 10** | | | |
| --- | --- | --- | --- |
| **Section** | **Q#** | **Data Extracted** | **Details** |
| **Study Information** | 1 | Study ID | Parent et al., 2018 |
|  | 2 | Title | Pilot project: Physiologic responses to a high-intensity active video game with COPD patients—Tools for home rehabilitation |
|  | 3 | Authors | Andrée-Anne Parent, Vincent Gosselin-Boucher, Marilyn Houle-Peloquin, Claude Poirier, Alain-Steve Comtois |
|  | 4 | Year of Publication | 2018 |
|  | 5 | Journal/Source | *Clinical Respiratory Journal* |
| **Study Design** | 6 | Study Type | Pilot feasibility study |
|  | 7 | Sample Size | N=14 |
|  | 8 | Study Setting | Supervised hospital setting in Montreal, Canada |
|  | 9 | Intervention Duration | Single session, 30 minutes |
| **Intervention Characteristics** | 10 | Type of Gamified Intervention | High-intensity exergames using motion capture (Kinect) |
|  | 11 | Platform/Technology Used | Kinect motion capture (Xbox One), Shape-Up game |
|  | 12 | Game Elements | Repetitive exercise mini-games (running, boxing, core twisting, squatting) with feedback |
|  | 13 | Tailoring/Personalization | Adjustments to exercise difficulty based on individual physical capacity |
|  | 14 | Integration with Healthcare | NA |
| **Participant Characteristics** | 15 | Population | COPD patients aged 69-74 years with moderate to severe COPD (FEV1 44% predicted) |
|  | 16 | Inclusion/Exclusion Criteria | COPD diagnosis; exclusion for cardiovascular or musculoskeletal conditions |
|  | 17 | Baseline Characteristics | FEV1: Men 37.4%, Women 52.8%; VC: 2.47 L |
| **Outcomes** | 18 | Primary Outcomes | Peak minute ventilation (36.8 L/min in squatting game), peak METs (4.4 in squatting game) |
|  | 19 | Secondary Outcomes | Reported enjoyment, motivation for home use, and exercise tolerance |
|  | 20 | Effectiveness Results | High-intensity games met exercise guidelines; Borg scores for leg exertion (13-14) |
|  | 21 | Behavioral Outcomes | High perceived enjoyment, willingness to engage in home-based rehabilitation |
|  | 22 | Engagement Metrics | 91% of participants reached high-intensity levels in squatting exercises |
|  | 23 | Quality of Life Improvements | NA |
| **Study Quality and Bias** | 24 | Risk of Bias Assessment | NA |
|  | 25 | Risk of Bias Findings | Moderate risk due to small sample size and single session |
|  | 26 | Limitations | Short study duration, small sample size, and no long-term follow-up |
| **Adaptation** | 27 | Cultural or Demographic Adaptation | NA |
|  | 28 | Technical Adaptation | None beyond Kinect customization |
|  | 29 | Challenges in Adaptation | Participants experienced some discomfort in using new technology |
| **Conclusions** | 30 | Study Conclusion | High-intensity video games are feasible for COPD rehabilitation, but more research is needed |
|  | 31 | Relevance to Gamified COPD Management | Highly relevant as a potential home-based COPD rehabilitation tool |
|  | 32 | Recommendations for Future Research | Need for long-term trials to assess adherence, safety, and effectiveness at home |

#### Appendix 3-11: Rutkowski et al., 2019

| **Study 11** | | | |
| --- | --- | --- | --- |
| **ection** | **Q#** | **Data Extracted** | **Details** |
| **Study Information** | 1 | Study ID | Rutkowski et al., 2019 |
|  | 2 | Title | Effect of virtual reality‐based rehabilitation on physical fitness in patients with COPD |
|  | 3 | Authors | Sebastian Rutkowski, Anna Rutkowska, Dariusz Jastrzębski, Henryk Racheniuk, Witold Pawełczyk, Jan Szczegielniak |
|  | 4 | Year of Publication | 2019 |
|  | 5 | Journal/Source | *Journal of Human Kinetics* |
| **Study Design** | 6 | Study Type | RCT |
|  | 7 | Sample Size | N=68 (EG: 34, CG: 34) |
|  | 8 | Study Setting | Hospital-based pulmonary rehabilitation (stationary) |
|  | 9 | Intervention Duration | 14 days |
| **Intervention Characteristics** | 10 | Type of Gamified Intervention | Virtual rehabilitation using Kinect-based motion training |
|  | 11 | Platform/Technology Used | Xbox 360 Kinect system, Kinect Adventures game |
|  | 12 | Game Elements | Avatar-based minigames involving rafting, ball hitting, roller-coaster riding |
|  | 13 | Tailoring/Personalization | Standard rehabilitation augmented with virtual reality exercises tailored for each patient |
|  | 14 | Integration with Healthcare | Integrated into a structured pulmonary rehabilitation program |
| **Participant Characteristics** | 15 | Population | 68 COPD patients (mean age 61.3 ± 3.7 years; GOLD stages B and C) |
|  | 16 | Inclusion/Exclusion Criteria | Inclusion: COPD stages B/C, age 50–70; Exclusion: severe comorbidities, cognitive disorders |
|  | 17 | Baseline Characteristics | Baseline SFT and spirometry; mean FEV1%pred: 64.15% |
| **Outcomes** | 18 | Primary Outcomes | Improved physical fitness as measured by SFT |
|  | 19 | Secondary Outcomes | Significant improvement in exercise tolerance (6MWT, Arm Curl, Chair Stand) |
|  | 20 | Effectiveness Results | Significant within-group improvements (*p*<0.05) in SFT tests (Arm Curl: 19 to 20.6, Chair Stand: 14.8 to 15.6) |
|  | 21 | Behavioral Outcomes | VR group showed enhanced motivation and adherence |
|  | 22 | Engagement Metrics | High adherence to both standard and virtual rehabilitation programs |
|  | 23 | Quality of Life Improvements | NA |
| **Study Quality and Bias** | 24 | Risk of Bias Assessment | Use of control group enhances reliability |
|  | 25 | Risk of Bias Findings | Moderate risk due to short intervention duration |
|  | 26 | Limitations | Short duration, lack of long-term follow-up, no blinding of participants |
| **Adaptation** | 27 | Cultural or Demographic Adaptation | NA |
|  | 28 | Technical Adaptation | Basic Kinect setup for stationary use; no advanced technical customizations |
|  | 29 | Challenges in Adaptation | Minor technical issues with Kinect system |
| **Conclusions** | 30 | Study Conclusion | VR-based rehabilitation is a feasible and effective addition to standard COPD rehabilitation |
|  | 31 | Relevance to Gamified COPD Management | High relevance; demonstrates potential for VR in physical rehabilitation for COPD patients |
|  | 32 | Recommendations for Future Research | Need for longer-term trials and home-based rehabilitation evaluation using low-cost VR systems |

#### Appendix 3-12: Sutanto et al., 2019

| **Study 12** | | | |
| --- | --- | --- | --- |
| **Section** | **Q#** | **Data Extracted** | **Details** |
| **Study Information** | 1 | Study ID | Sutanto et al., 2019 |
|  | 2 | Title | Videogame assisted exercise training in patients with chronic obstructive pulmonary disease: A preliminary study |
|  | 3 | Authors | Y.S. Sutanto, D.N. Makhabah, J. Aphridasari, M. Doewes, Suradi, N. Ambrosino |
|  | 4 | Year of Publication | 2019 |
|  | 5 | Journal/Source | *Pulmonology* |
| **Study Design** | 6 | Study Type | RCT |
|  | 7 | Sample Size | N=23 (EG: 10, CG: 10, 3 dropouts) |
|  | 8 | Study Setting | Outpatient clinic in Dr. Moewardi Hospital, Surakarta, Indonesia |
|  | 9 | Intervention Duration | 6 weeks |
| **Intervention Characteristics** | 10 | Type of Gamified Intervention | Wii Fit-based exercise training |
|  | 11 | Platform/Technology Used | Wii Fit balance board and TV system |
|  | 12 | Game Elements | Yoga, strength training, aerobic exercises, feedback system, and virtual trainer |
|  | 13 | Tailoring/Personalization | Individualized program with game duration, difficulty, and scores recorded |
|  | 14 | Integration with Healthcare | Integrated into a hospital-based outpatient exercise program |
| **Participant Characteristics** | 15 | Population | COPD patients (mean age 65.1 ± 7.5 years; GOLD stages C and D) |
|  | 16 | Inclusion/Exclusion Criteria | Inclusion: COPD patients with stable conditions; Exclusion: acute exacerbation, severe comorbidities |
|  | 17 | Baseline Characteristics | Mean FEV1% predicted: 49.1%, BMI: 19.3 kg/m² |
| **Outcomes** | 18 | Primary Outcomes | 6MWD, dyspnea (TDI), and health-related quality of life (SGRQ) |
|  | 19 | Secondary Outcomes | BODE index, MRC scale |
|  | 20 | Effectiveness Results | 6MWD improved significantly (EG: 376.6 to 420m; CG: 410.7 to 477.5m, *p*=0.0001) |
|  | 21 | Behavioral Outcomes | NA |
|  | 22 | Engagement Metrics | High adherence to the Wii Fit program |
|  | 23 | Quality of Life Improvements | Significant SGRQ score reduction in both groups (EG: 57.7 to 30.6, CG: 54.1 to 29.4) |
| **Study Quality and Bias** | 24 | Risk of Bias Assessment | NA |
|  | 25 | Risk of Bias Findings | Moderate risk due to small sample size |
|  | 26 | Limitations | Small sample size, unblinded study, lack of intensity monitoring for the Wii exercises |
| **Adaptation** | 27 | Cultural or Demographic Adaptation | Conducted in an Indonesian context |
|  | 28 | Technical Adaptation | Wii Fit program customized to the local setting, no major technical challenges |
|  | 29 | Challenges in Adaptation | Limited intensity tracking, high cost of the Wii Fit program |
| **Conclusions** | 30 | Study Conclusion | Wii Fit exercise training is feasible but did not provide additional benefit over standard training |
|  | 31 | Relevance to Gamified COPD Management | High relevance as a potential home-based rehabilitation tool |
|  | 32 | Recommendations for Future Research | Larger trials needed, assess cost-effectiveness of Wii Fit in multidisciplinary rehab programs |

#### Appendix 3-13: Jung et al., 2020

| **Study 13** | | | |
| --- | --- | --- | --- |
| **Section** | **Q#** | **Data Extracted** | **Details** |
| **Study Information** | 1 | Study ID | Jung et al., 2020 |
|  | 2 | Title | A virtual reality–supported intervention for pulmonary rehabilitation of patients with chronic obstructive pulmonary disease: Mixed methods study |
|  | 3 | Authors | Timothy Jung, Natasha Moorhouse, Xin Shi, Muhammad Farhan Amin |
|  | 4 | Year of Publication | 2020 |
|  | 5 | Journal/Source | *Journal of Medical Internet Research (JMIR)* |
| **Study Design** | 6 | Study Type | Mixed methods study |
|  | 7 | Sample Size | N=10 |
|  | 8 | Study Setting | South and West Cumbria, UK, home-based rehabilitation |
|  | 9 | Intervention Duration | 8 weeks |
| **Intervention Characteristics** | 10 | Type of Gamified Intervention | Virtual reality-supported pulmonary rehabilitation |
|  | 11 | Platform/Technology Used | VR headset (Pico Goblin), PR in VR app |
|  | 12 | Game Elements | 3D avatars, educational modules, immersive experience |
|  | 13 | Tailoring/Personalization | Exercises tailored to COPD patient’s severity level (MRC 4 or 5) |
|  | 14 | Integration with Healthcare | Real-time remote monitoring of heart rate and oxygen saturation |
| **Participant Characteristics** | 15 | Population | Elderly COPD patients (MRC 4 or 5), aged 63-75 |
|  | 16 | Inclusion/Exclusion Criteria | Inclusion: COPD (MRC 4 or 5), exclusion not explicitly detailed |
|  | 17 | Baseline Characteristics | MRC scale, self-reported measures for anxiety, depression, physical function |
| **Outcomes** | 18 | Primary Outcomes | Improved compliance, physical health (mobility, flexibility), psychological well-being |
|  | 19 | Secondary Outcomes | Improved quality of life, patient satisfaction, engagement |
|  | 20 | Effectiveness Results | Significant improvement in patient’s physical function, reduced anxiety and depression |
|  | 21 | Behavioral Outcomes | Increased confidence and motivation to exercise |
|  | 22 | Engagement Metrics | High engagement due to enjoyment and immersive aspects |
|  | 23 | Quality of Life Improvements | Improved self-reported health-related quality of life (HRQoL) |
| **Study Quality and Bias** | 24 | Risk of Bias Assessment | NA |
|  | 25 | Risk of Bias Findings | Small sample size; acknowledged limitations |
|  | 26 | Limitations | Small sample size, limited generalizability |
| **Adaptation** | 27 | Cultural or Demographic Adaptation | NA |
|  | 28 | Technical Adaptation | Feedback on improving headset weight and app functionality |
|  | 29 | Challenges in Adaptation | Minor technical glitches; request for more customizable exercise levels |
| **Conclusions** | 30 | Study Conclusion | VR-based rehabilitation increased compliance, enhanced physical and mental well-being, offering a feasible alternative to traditional pulmonary rehabilitation |
|  | 31 | Relevance to Gamified COPD Management | High relevance, demonstrated effectiveness of VR for pulmonary rehabilitation |
|  | 32 | Recommendations for Future Research | Larger sample size, explore long-term effects, further development of technology |

#### Appendix 3-14: Rutkowski et al., 2020

| **Study 14** | | | |
| --- | --- | --- | --- |
| **Section** | **Q#** | **Data Extracted** | **Details** |
| **Study Information** | 1 | Study ID | Rutkowski et al., 2020 |
|  | 2 | Title | Virtual Reality Rehabilitation in Patients with Chronic Obstructive Pulmonary Disease: A Randomized Controlled Trial |
|  | 3 | Authors | Sebastian Rutkowski, Anna Rutkowska, Paweł Kiper, Dariusz Jastrzebski, Henryk Racheniuk, Andrea Turolla, Jan Szczegielniak, Richard Casaburi |
|  | 4 | Year of Publication | 2020 |
|  | 5 | Journal/Source | *International Journal of Chronic Obstructive Pulmonary Disease* |
| **Study Design** | 6 | Study Type | RCT |
|  | 7 | Sample Size | N=106 (34 ET, 38 ET+VR, 34 VR) |
|  | 8 | Study Setting | Specialist Hospital in Głuchołazy, Poland |
|  | 9 | Intervention Duration | 2 weeks |
| **Intervention Characteristics** | 10 | Type of Gamified Intervention | Virtual reality-based rehabilitation using Xbox Kinect and Kinect Adventures software |
|  | 11 | Platform/Technology Used | Xbox 360, Kinect, Kinect Adventures |
|  | 12 | Game Elements | Mini-games involving rafting, ball-hitting, dynamic balance, and coordination |
|  | 13 | Tailoring/Personalization | Exercises adapted to patients’ abilities (age, physical condition) |
|  | 14 | Integration with Healthcare | Supervised by physiotherapists; heart rate monitored |
| **Participant Characteristics** | 15 | Population | COPD patients aged 50–70, GOLD stages 2 and 3 |
|  | 16 | Inclusion/Exclusion Criteria | Inclusion: COPD stages 2/3, 50–70 years; Exclusion: pneumonia, heart failure, uncontrolled diabetes, cognitive disorders |
|  | 17 | Baseline Characteristics | Mean FEV1%pred (ET: 65.4%, ET+VR: 60.5%, VR: 69.2%) |
| **Outcomes** | 18 | Primary Outcomes | Significant improvement in Senior Fitness Test (Arm curl, Chair stand, 6MWT) |
|  | 19 | Secondary Outcomes | Flexibility (Back scratch, Chair sit and reach), agility (Up and go) |
|  | 20 | Effectiveness Results | ET+VR superior to ET (e.g., 6MWT: ET+VR: +39.11m, ET: +16.24m) |
|  | 21 | Behavioral Outcomes | Enhanced motivation and adherence in VR-based exercises |
|  | 22 | Engagement Metrics | High adherence (95% participation rate) |
|  | 23 | Quality of Life Improvements | NA |
| **Study Quality and Bias** | 24 | Risk of Bias Assessment | Randomization was applied |
|  | 25 | Risk of Bias Findings | Low risk due to structured randomization and control group |
|  | 26 | Limitations | Short duration (2 weeks), only GOLD stages 2 and 3 included |
| **Adaptation** | 27 | Cultural or Demographic Adaptation | NA |
|  | 28 | Technical Adaptation | None beyond basic setup with Kinect |
|  | 29 | Challenges in Adaptation | None significant; minor technical adjustments needed |
| **Conclusions** | 30 | Study Conclusion | VR-based rehabilitation shows greater improvement in physical fitness than traditional ET alone; viable addition to pulmonary rehab |
|  | 31 | Relevance to Gamified COPD Management | Highly relevant due to VR application for improving COPD management |
|  | 32 | Recommendations for Future Research | Longer trials with outpatient settings and quality of life assessments needed |

#### Appendix 3-15: Tu et al., 2020

| **Study 15** | | | |
| --- | --- | --- | --- |
| **Section** | **Q#** | **Data Extracted** | **Details** |
| **Study Information** | 1 | Study ID | Tu et al., 2020 |
|  | 2 | Title | BreathCoach: A Smart In-home Breathing Training System with Bio-feedback via VR Game |
|  | 3 | Authors | Linlin Tu, Tian Hao, Chongguang Bi, Guoliang Xing |
|  | 4 | Year of Publication | 2020 |
|  | 5 | Journal/Source | *Smart Health* |
| **Study Design** | 6 | Study Type | Pilot feasibility study |
|  | 7 | Sample Size | Proof-of-concept demo; no specific patient sample size indicated |
|  | 8 | Study Setting | Home-based (in-lab demo using smartwatch, smartphone, and VR headset) |
|  | 9 | Intervention Duration | Demo sessions lasted 2-5 minutes |
| **Intervention Characteristics** | 10 | Type of Gamified Intervention | VR-assisted biofeedback breathing training using RSA-BT |
|  | 11 | Platform/Technology Used | Smartphone, smartwatch (Empatica E4), VR viewer (Google Cardboard) |
|  | 12 | Game Elements | Breathing control, interactive VR environments, avatars, real-time biofeedback |
|  | 13 | Tailoring/Personalization | Dynamic adjustment of breathing patterns based on real-time physiological data |
|  | 14 | Integration with Healthcare | No direct integration with healthcare, but relevant for cardiorespiratory rehabilitation |
| **Participant Characteristics** | 15 | Population | Not explicitly mentioned, but targeted at patients needing RSA-BT for anxiety or respiratory conditions |
|  | 16 | Inclusion/Exclusion Criteria | NA |
|  | 17 | Baseline Characteristics | Not applicable in demo phase |
| **Outcomes** | 18 | Primary Outcomes | Feasibility of smart in-home breathing training with RSA-BT |
|  | 19 | Secondary Outcomes | User feedback on usability, engagement, and real-time performance improvements |
|  | 20 | Effectiveness Results | Real-time biofeedback effectively guided breathing patterns |
|  | 21 | Behavioral Outcomes | Improved engagement with breathing exercises due to immersive VR |
|  | 22 | Engagement Metrics | High engagement in demo sessions; real-time feedback kept users on track |
|  | 23 | Quality of Life Improvements | NA |
| **Study Quality and Bias** | 24 | Risk of Bias Assessment | NA |
|  | 25 | Risk of Bias Findings | NA |
|  | 26 | Limitations | Small-scale demo, short duration, no long-term data |
| **Adaptation** | 27 | Cultural or Demographic Adaptation | NA |
|  | 28 | Technical Adaptation | Uses lightweight algorithms and readily available devices for home use |
|  | 29 | Challenges in Adaptation | Some technical refinements (e.g., headset comfort, sound effects) suggested by users |
| **Conclusions** | 30 | Study Conclusion | BreathCoach shows feasibility for in-home breathing training with real-time feedback in VR |
|  | 31 | Relevance to Gamified COPD Management | High relevance for gamified COPD management through VR-based breathing exercises |
|  | 32 | Recommendations for Future Research | Further testing with larger samples and development of more game designs and sound features |

#### Appendix 3-16: Rutkowski et al., 2021

| **Study 16** | | | |
| --- | --- | --- | --- |
| **Section** | **Q#** | **Data Extracted** | **Details** |
| **Study Information** | 1 | Study ID | Rutkowski et al., 2021 |
|  | 2 | Title | Evaluation of the Efficacy of Immersive Virtual Reality Therapy as a Method Supporting Pulmonary Rehabilitation |
|  | 3 | Authors | Sebastian Rutkowski, Jan Szczegielniak, Joanna Szczepańska-Gieracha |
|  | 4 | Year of Publication | 2021 |
|  | 5 | Journal/Source | *Journal of Clinical Medicine* |
| **Study Design** | 6 | Study Type | Randomized Controlled Trial |
|  | 7 | Sample Size | N=50 |
|  | 8 | Study Setting | Specialist Hospital, Głuchołazy, Poland |
|  | 9 | Intervention Duration | 2 weeks (10 VR sessions) |
| **Intervention Characteristics** | 10 | Type of Gamified Intervention | Immersive virtual reality therapy |
|  | 11 | Platform/Technology Used | VR TierOne device (head-mounted display) |
|  | 12 | Game Elements | Virtual therapeutic garden, metaphoric health recovery |
|  | 13 | Tailoring/Personalization | Emotional balance recovery and mood improvement tailored to patient recovery |
|  | 14 | Integration with Healthcare | Supervised by therapists as part of pulmonary rehabilitation |
| **Participant Characteristics** | 15 | Population | COPD patients aged 45-85 with anxiety or depressive symptoms |
|  | 16 | Inclusion/Exclusion Criteria | Inclusion: COPD, anxiety or depressive symptoms score > 8 on HADS; Exclusion: cognitive impairment, psychotic symptoms |
|  | 17 | Baseline Characteristics | FEV1%pred, 6MWT, HADS for anxiety, depression |
| **Outcomes** | 18 | Primary Outcomes | Reduction in emotional tension (*p* < 0.0003), external stress (*p* < 0.0092), depression (*p* < 0.0001), anxiety (*p* < 0.0009) |
|  | 19 | Secondary Outcomes | Functional capacity improvement (6MWT, *p* = 0.0018 for VR group) |
|  | 20 | Effectiveness Results | VR group showed significant stress, anxiety, and depression reduction compared to control |
|  | 21 | Behavioral Outcomes | Increased mood and emotional balance through immersive therapy |
|  | 22 | Engagement Metrics | High engagement in VR group with full participation over the 2 weeks |
|  | 23 | Quality of Life Improvements | Statistically significant improvements in psychological well-being |
| **Study Quality and Bias** | 24 | Risk of Bias Assessment | Assessor-blinded RCT with controlled randomization (low risk of bias) |
|  | 25 | Risk of Bias Findings | Low risk due to structured randomization and control group |
|  | 26 | Limitations | Short duration, only hospital-based |
| **Adaptation** | 27 | Cultural or Demographic Adaptation | NA |
|  | 28 | Technical Adaptation | Use of VR TierOne device, simple immersion setup |
|  | 29 | Challenges in Adaptation | NA |
| **Conclusions** | 30 | Study Conclusion | VR therapy enhances traditional pulmonary rehabilitation by improving mood, reducing anxiety and stress in COPD patients |
|  | 31 | Relevance to Gamified COPD Management | Highly relevant for enhancing mood and emotional well-being through virtual environments in COPD rehabilitation |
|  | 32 | Recommendations for Future Research | Longer-term studies, exploring post-rehabilitation impacts, and adding objective stress measures like cortisol levels |

#### Appendix 3-17: Simmich et al., 2021

| **Study 17** | | | |
| --- | --- | --- | --- |
| **Section** | **Q#** | **Data Extracted** | **Details** |
| **Study Information** | 1 | Study ID | Simmich et al., 2021 |
|  | 2 | Title | Perspectives of older adults with chronic disease on the use of wearable technology and video games for physical activity |
|  | 3 | Authors | Joshua Simmich, Allison Mandrusiak, Trevor Russell, Stuart Smith, Nicole Hartley |
|  | 4 | Year of Publication | 2021 |
|  | 5 | Journal/Source | *Digital Health* |
| **Study Design** | 6 | Study Type | Qualitative study (semi-structured interviews) |
|  | 7 | Sample Size | N=19 |
|  | 8 | Study Setting | Pulmonary support groups in Brisbane, Australia |
|  | 9 | Intervention Duration | NA (single interview session per participant) |
| **Intervention Characteristics** | 10 | Type of Gamified Intervention | AVGs as a rehabilitation tool were discussed |
|  | 11 | Platform/Technology Used | Wearable activity trackers, smartphones, AVGs like Wii, Xbox Kinect |
|  | 12 | Game Elements | General perceptions of AVGs and wearable trackers were explored |
|  | 13 | Tailoring/Personalization | NA |
|  | 14 | Integration with Healthcare | Participants were interested in sharing data with clinicians for feedback and improved clinical care |
| **Participant Characteristics** | 15 | Population | Older adults with chronic obstructive pulmonary disease (COPD), mean age 70 years, 58% female |
|  | 16 | Inclusion/Exclusion Criteria | Diagnosis of COPD, experience with formal exercise programs, ability to exercise independently |
|  | 17 | Baseline Characteristics | Disease severity assessed using the Medical Research Council (MRC) dyspnea scale and the CCQ |
| **Outcomes** | 18 | Primary Outcomes | Perceptions of wearables and AVGs as tools for rehabilitation |
|  | 19 | Secondary Outcomes | Barriers and motivators for using wearables and AVGs (e.g., perceived usefulness, enjoyment, goal-setting) |
|  | 20 | Effectiveness Results | Participants found wearable trackers useful for quantifying activity, setting goals, and tracking improvements over time |
|  | 21 | Behavioral Outcomes | AVGs were seen as fun and motivating for physical activity, but some participants felt they were too difficult or not beneficial |
|  | 22 | Engagement Metrics | Participants’ interest in wearables increased with social interaction and family involvement; challenges in long-term adherence were noted |
|  | 23 | Quality of Life Improvements | No specific tools used to measure quality of life, but general health benefits of physical activity were discussed |
| **Study Quality and Bias** | 24 | Risk of Bias Assessment | NA |
|  | 25 | Risk of Bias Findings | Low risk (small sample size, self-reported data) |
|  | 26 | Limitations | Lack of generalizability due to the small sample size and limited geographical representation |
| **Adaptation** | 27 | Cultural or Demographic Adaptation | NA |
|  | 28 | Technical Adaptation | NA |
|  | 29 | Challenges in Adaptation | Participants struggled with technology complexity and preferred more straightforward options |
| **Conclusions** | 30 | Study Conclusion | Wearable technology and AVGs could help older adults with COPD engage in physical activity, but ease of use and clinician involvement are crucial for long-term adherence |
|  | 31 | Relevance to Gamified COPD Management | High relevance, as the study discusses the use of wearables and AVGs in managing physical activity in COPD patients |
|  | 32 | Recommendations for Future Research | Future studies should explore long-term adherence to wearable technology and AVGs and examine ways to integrate them into clinical care |

#### Appendix 3-18: Simmich et al., 2021

| **Study 18** | | | |
| --- | --- | --- | --- |
| **Section** | **Q#** | **Data Extracted** | **Details** |
| **Study Information** | 1 | Study ID | Simmich et al., 2021 |
|  | 2 | Title | A Co-Designed Active Video Game for Physical Activity Promotion in People With Chronic Obstructive Pulmonary Disease: Pilot Trial |
|  | 3 | Authors | Joshua Simmich, Allison Mandrusiak, Stuart Trevor Smith, Nicole Hartley, Trevor Glen Russell |
|  | 4 | Year of Publication | 2021 |
|  | 5 | Journal/Source | *JMIR Serious Games* |
| **Study Design** | 6 | Study Type | Pilot RCT |
|  | 7 | Sample Size | N=18 (EG: 9; CG: 9) |
|  | 8 | Study Setting | Home-based intervention, Queensland, Australia |
|  | 9 | Intervention Duration | 3 weeks |
| **Intervention Characteristics** | 10 | Type of Gamified Intervention | AVG focusing on physical activities |
|  | 11 | Platform/Technology Used | Smartphone app with Fitbit integration |
|  | 12 | Game Elements | Single-player and multiplayer modes, progress tracking, rewards for completing exercises |
|  | 13 | Tailoring/Personalization | Players selected difficulty levels for each exercise |
|  | 14 | Integration with Healthcare | Clinicians monitored progress via a web interface |
| **Participant Characteristics** | 15 | Population | COPD patients, mean age 70 (experiment group) and 65 (control group), mostly retired |
|  | 16 | Inclusion/Exclusion Criteria | COPD diagnosis, attended pulmonary rehabilitation, able to exercise independently |
|  | 17 | Baseline Characteristics | Moderate functional limitations, mean MRC dyspnea score of 2.4 |
| **Outcomes** | 18 | Primary Outcomes | Usage of the game (58.6% of days logged), daily steps, MVPA |
|  | 19 | Secondary Outcomes | Engagement metrics (IMI, GEQ, CPCA), adherence to Fitbit |
|  | 20 | Effectiveness Results | 9 min/day increase in MVPA (experiment group), 2% decrease in steps (experiment) vs 13% decrease (control) |
|  | 21 | Behavioral Outcomes | Positive correlation between game usage and steps, weak correlation with MVPA |
|  | 22 | Engagement Metrics | High adherence to Fitbit (84.3% of days), moderate GEQ score of 30.4 |
|  | 23 | Quality of Life Improvements | No significant improvements reported |
| **Study Quality and Bias** | 24 | Risk of Bias Assessment | NA |
|  | 25 | Risk of Bias Findings | Possible bias due to co-design involvement of experiment group |
|  | 26 | Limitations | Small sample size, short trial duration, Fitbit issues, lack of notifications in the game |
| **Adaptation** | 27 | Cultural or Demographic Adaptation | NA |
|  | 28 | Technical Adaptation | Limiting engagement |
|  | 29 | Challenges in Adaptation | Bluetooth synchronization issues with Fitbit |
| **Conclusions** | 30 | Study Conclusion | AVG shows promise for maintaining physical activity post-rehabilitation; further testing required |
|  | 31 | Relevance to Gamified COPD Management | Relevant as a tool for sustaining physical activity and engagement with wearable technology |
|  | 32 | Recommendations for Future Research | Larger trials, longer follow-up, improved notification systems in the app |

#### Appendix 3-19: Baxter et al., 2022

| **Study 19** | | | |
| --- | --- | --- | --- |
| **Section** | **Q#** | **Data Extracted** | **Details** |
| **Study Information** | 1 | Study ID | Baxter et al., 2022 |
|  | 2 | Title | Virtual respiratory therapy delivered through a smartphone app: A mixed-methods randomised usability study |
|  | 3 | Authors | Clarence Anthony Baxter, Julie-Anne Carroll, Brendan Keogh, Corneel Vandelanotte |
|  | 4 | Year of Publication | 2022 |
|  | 5 | Journal/Source | *BMJ Open Respiratory Research* |
| **Study Design** | 6 | Study Type | Mixed-methods randomised usability study |
|  | 7 | Sample Size | N=24 |
|  | 8 | Study Setting | Urban locations in South-East Queensland, Australia |
|  | 9 | Intervention Duration | Single session (three inspirations using each device) |
| **Intervention Characteristics** | 10 | Type of Gamified Intervention | Virtual incentive spirometry via smartphone app |
|  | 11 | Platform/Technology Used | QUT Inspire app, smartphones (Apple/Android) |
|  | 12 | Game Elements | Visual rewards, breath timer, inspiration counter |
|  | 13 | Tailoring/Personalization | Adjustable microphone sensitivity, text or video instructions |
|  | 14 | Integration with Healthcare | No direct integration with clinical care at this stage |
| **Participant Characteristics** | 15 | Population | Healthy adults, mean age 39.2 years (range 21-64), 58% female |
|  | 16 | Inclusion/Exclusion Criteria | Exclusion: medical conditions preventing maximal inspirations |
|  | 17 | Baseline Characteristics | Varied smartphone familiarity; 70.8% had no prior ISy experience |
| **Outcomes** | 18 | Primary Outcomes | Comparable inspiration durations between QUT Inspire (7.3±2.0s) and Triflo II (7.5±2.3s) |
|  | 19 | Secondary Outcomes | User satisfaction with app’s usability, responsiveness, and animations |
|  | 20 | Effectiveness Results | No significant differences in usability or performance between the app and the clinical device |
|  | 21 | Behavioral Outcomes | Some users preferred app due to less perceived inspiratory effort |
|  | 22 | Engagement Metrics | High satisfaction with visual rewards; 75% found the timer motivating |
|  | 23 | Quality of Life Improvements | NA |
| **Study Quality and Bias** | 24 | Risk of Bias Assessment | Not explicitly stated, but randomisation was used |
|  | 25 | Risk of Bias Findings | Low risk of bias due to randomisation and crossover design |
|  | 26 | Limitations | Small sample, short session duration, no clinical participants |
| **Adaptation** | 27 | Cultural or Demographic Adaptation | NA |
|  | 28 | Technical Adaptation | Distance measurement for inspiratory detection needed improvement |
|  | 29 | Challenges in Adaptation | App required further technical refinement to improve microphone sensitivity |
| **Conclusions** | 30 | Study Conclusion | The virtual ISy app was effective, user-friendly, and preferred for its portability and responsiveness |
|  | 31 | Relevance to Gamified COPD Management | Relevant for potential integration into respiratory rehabilitation for chronic conditions like COPD |
|  | 32 | Recommendations for Future Research | Further studies in clinical settings and enhancements to app functionality (e.g., better proximity sensors) |

#### Appendix 3-20: Oberschmidt et al., 2022

| **Study 20** | | | |
| --- | --- | --- | --- |
| **Section** | **Q#** | **Data Extracted** | **Details** |
| **Study Information** | 1 | Study ID | Oberschmidt et al., 2022 |
|  | 2 | Title | Patient Values Associated with an Exergame Supporting COPD Treatment |
|  | 3 | Authors | Kira Oberschmidt, Marijke Broekhuis, Christiane Grünloh |
|  | 4 | Year of Publication | 2022 |
|  | 5 | Journal/Source | *Studies in Health Technology and Informatics* |
| **Study Design** | 6 | Study Type | Qualitative study using interviews |
|  | 7 | Sample Size | N=7 (2 completed the 6-month study) |
|  | 8 | Study Setting | Physiotherapy office in the Netherlands |
|  | 9 | Intervention Duration | 6 months |
| **Intervention Characteristics** | 10 | Type of Gamified Intervention | Exergame used as part of physiotherapy treatment |
|  | 11 | Platform/Technology Used | TV screen with motion-sensing camera for exercise tracking |
|  | 12 | Game Elements | Audio-visual feedback during exercises, score tracking |
|  | 13 | Tailoring/Personalization | Feedback based on exercise accuracy, adjustable difficulty levels |
|  | 14 | Integration with Healthcare | Integrated into routine physiotherapy treatment |
| **Participant Characteristics** | 15 | Population | COPD patients, aged 55–74, 5 males, moderate to severe COPD |
|  | 16 | Inclusion/Exclusion Criteria | COPD patients undergoing physiotherapy |
|  | 17 | Baseline Characteristics | Participants had been in physiotherapy for 4-8 years; mostly moderate GOLD stage 2 |
| **Outcomes** | 18 | Primary Outcomes | Key patient values identified: Independence, Personal Guidance, Trust, Regularity |
|  | 19 | Secondary Outcomes | Changes in value prioritization over time |
|  | 20 | Effectiveness Results | Exergames supported values like Independence and Challenge, but hindered Personal Guidance and Social Interaction |
|  | 21 | Behavioral Outcomes | Independence valued, but personal support needed when using exergames |
|  | 22 | Engagement Metrics | Exergames promoted challenge and seeing results, motivating participants |
|  | 23 | Quality of Life Improvements | Participants valued seeing progress in their treatment, but no formal QoL metrics were reported |
| **Study Quality and Bias** | 24 | Risk of Bias Assessment | NA |
|  | 25 | Risk of Bias Findings | Some dropouts due to exacerbation, but not directly related to intervention |
|  | 26 | Limitations | Small sample size, dropouts after 12 weeks, and occasional technical issues |
| **Adaptation** | 27 | Cultural or Demographic Adaptation | NA |
|  | 28 | Technical Adaptation | Issues with camera accuracy during exercise detection |
|  | 29 | Challenges in Adaptation | Technical errors with exercise detection and loud notifications disrupted patient comfort |
| **Conclusions** | 30 | Study Conclusion | Exergames support COPD patients’ values like Independence and Regularity but may hinder Social Interaction and Personal Guidance |
|  | 31 | Relevance to Gamified COPD Management | Highly relevant for understanding patient values in gamified interventions for COPD |
|  | 32 | Recommendations for Future Research | Further research on balancing values like Independence with Social Interaction in exergames is needed |

#### Appendix 3-21: Finkelstein et al., 2023

| **Study 21** | | | |
| --- | --- | --- | --- |
| **Section** | **Q#** | **Data Extracted** | **Details** |
| **Study Information** | 1 | Study ID | Finkelstein et al., 2023 |
|  | 2 | Title | Feasibility of a Virtual Reality App to Promote Pulmonary Rehabilitation |
|  | 3 | Authors | Joseph Finkelstein, Irena Parvanova, Xingye Huo |
|  | 4 | Year of Publication | 2023 |
|  | 5 | Journal/Source | *Studies in Health Technology and Informatics* |
| **Study Design** | 6 | Study Type | Mixed-methods randomized usability study |
|  | 7 | Sample Size | N=9 |
|  | 8 | Study Setting | Icahn School of Medicine at Mount Sinai, New York, USA |
|  | 9 | Intervention Duration | Single session of using the VR app |
| **Intervention Characteristics** | 10 | Type of Gamified Intervention | Virtual reality educational app for pulmonary rehabilitation (PR) |
|  | 11 | Platform/Technology Used | Oculus Quest 2 with VR headset and controllers |
|  | 12 | Game Elements | Interactive educational modules, multiple-choice questions, visual feedback |
|  | 13 | Tailoring/Personalization | Simplified user interface with preset controls for ease of use |
|  | 14 | Integration with Healthcare | Not integrated with clinical care; focused on patient education |
| **Participant Characteristics** | 15 | Population | 9 COPD patients, 67% female, aged 59-82 years |
|  | 16 | Inclusion/Exclusion Criteria | COPD diagnosis, no prior VR experience |
|  | 17 | Baseline Characteristics | Marginal health literacy (mean score of 16.2 on BRIEF Health Literacy Screening Tool) |
| **Outcomes** | 18 | Primary Outcomes | High usability and user acceptance (mean SUS score: 95.8) |
|  | 19 | Secondary Outcomes | Significant increase in PR knowledge (mean score increase from 7.2 to 7.9, *p*<0.04) |
|  | 20 | Effectiveness Results | 89% of participants successfully completed the first task, and 100% completed tasks 2 and 3 without prompts |
|  | 21 | Behavioral Outcomes | High interest in using VR for patient empowerment and PR education |
|  | 22 | Engagement Metrics | Positive feedback for visual feedback, ease of navigation, and VR app structure |
|  | 23 | Quality of Life Improvements | NA |
| **Study Quality and Bias** | 24 | Risk of Bias Assessment | NA |
|  | 25 | Risk of Bias Findings | Low risk; all participants completed the tasks without significant issues |
|  | 26 | Limitations | Small sample size, lack of long-term follow-up |
| **Adaptation** | 27 | Cultural or Demographic Adaptation | NA |
|  | 28 | Technical Adaptation | Simplified controls and interface for older adults with limited computer skills |
|  | 29 | Challenges in Adaptation | Minor difficulties in finding and starting the app initially |
| **Conclusions** | 30 | Study Conclusion | VR-based education app is feasible and well-accepted, significantly improving PR knowledge |
|  | 31 | Relevance to Gamified COPD Management | Highly relevant as an educational tool to promote PR and patient engagement |
|  | 32 | Recommendations for Future Research | Further development of VR apps for patient engagement and long-term studies to assess clinical outcomes |

#### Appendix 3-22: Gabriel et al., 2023

| **Study 22** | | | |
| --- | --- | --- | --- |
| **Section** | **Q#** | **Data Extracted** | **Details** |
| **Study Information** | 1 | Study ID | Gabriel et al., 2023 |
|  | 2 | Title | Mixed-Methods Assessment of a Virtual Reality-Based System for Pulmonary Rehabilitation |
|  | 3 | Authors | Aileen S. Gabriel, Te-Yi Tsai, Taulant Xhakli, Joseph Finkelstein |
|  | 4 | Year of Publication | 2023 |
|  | 5 | Journal/Source | *Studies in Health Technology and Informatics* |
| **Study Design** | 6 | Study Type | Mixed-methods usability study |
|  | 7 | Sample Size | N=18 (EG: 9; CG: 9) |
|  | 8 | Study Setting | Icahn School of Medicine at Mount Sinai, New York, USA |
|  | 9 | Intervention Duration | Single session using the VR app |
| **Intervention Characteristics** | 10 | Type of Gamified Intervention | VR-based system for pulmonary rehabilitation (education and exercises) |
|  | 11 | Platform/Technology Used | Oculus Quest 2, VR headset, and controllers |
|  | 12 | Game Elements | Educational modules, multiple-choice quizzes, guided exercises with visual instructions |
|  | 13 | Tailoring/Personalization | Simplified interface, single-button navigation, custom instructions for each user |
|  | 14 | Integration with Healthcare | No direct healthcare integration, but designed for patient engagement in PR at home |
| **Participant Characteristics** | 15 | Population | COPD patients, aged 55-84 (mean 74 in education group, 72 in exercise group) |
|  | 16 | Inclusion/Exclusion Criteria | COPD patients, recent history of acute exacerbation, no prior VR experience |
|  | 17 | Baseline Characteristics | Health literacy scores assessed (BRIEF Health Literacy Tool), no prior VR use |
| **Outcomes** | 18 | Primary Outcomes | High usability scores (System Usability Scale: 95.8/100) |
|  | 19 | Secondary Outcomes | High acceptance of the VR app, positive feedback on ease of navigation and content clarity |
|  | 20 | Effectiveness Results | Successful completion of PR tasks by all participants with minimal guidance |
|  | 21 | Behavioral Outcomes | Increased willingness to engage with home-based PR through VR |
|  | 22 | Engagement Metrics | High satisfaction with visual feedback and educational content (mean post-task scores: 4.74–4.89/5) |
|  | 23 | Quality of Life Improvements | NA |
| **Study Quality and Bias** | 24 | Risk of Bias Assessment | NA |
|  | 25 | Risk of Bias Findings | Low risk of bias due to comprehensive task completion by all participants |
|  | 26 | Limitations | Small sample size, no control group, lack of long-term follow-up |
| **Adaptation** | 27 | Cultural or Demographic Adaptation | NA |
|  | 28 | Technical Adaptation | Simplified interface and navigation for older adults with limited tech experience |
|  | 29 | Challenges in Adaptation | Minor difficulties in initial navigation and setup |
| **Conclusions** | 30 | Study Conclusion | VR-based pulmonary rehabilitation is feasible and highly accepted, supporting further development for home-based PR |
|  | 31 | Relevance to Gamified COPD Management | Highly relevant, offering immersive solutions for enhancing COPD rehabilitation |
|  | 32 | Recommendations for Future Research | Future studies should include control groups, long-term follow-up, and explore clinical impacts |

#### Appendix 3-23: Gabriel et al., 2023

| **Study 23** | | | |
| --- | --- | --- | --- |
| **Section** | **Q#** | **Data Extracted** | **Details** |
| **Study Information** | 1 | Study ID | Gabriel et al., 2023 |
|  | 2 | Title | Patient Perceptions of a Virtual Reality-Based System for Pulmonary Rehabilitation: A Qualitative Analysis |
|  | 3 | Authors | Aileen S. Gabriel, Te-Yi Tsai, Taulant Xhakli, Joseph Finkelstein |
|  | 4 | Year of Publication | 2023 |
|  | 5 | Journal/Source | Studies in Health Technology and Informatics |
| **Study Design** | 6 | Study Type | Qualitative study with semi-structured interviews |
|  | 7 | Sample Size | N=9 |
|  | 8 | Study Setting | Home-based, conducted virtually |
|  | 9 | Intervention Duration | Single session |
| **Intervention Characteristics** | 10 | Type of Gamified Intervention | VR-based exercise app for pulmonary rehabilitation |
|  | 11 | Platform/Technology Used | VR headset and controllers |
|  | 12 | Game Elements | Interactive guided exercises, visual feedback |
|  | 13 | Tailoring/Personalization | Simplified controls, visual guidance for exercises |
|  | 14 | Integration with Healthcare | Not directly integrated; focused on self-management |
| **Participant Characteristics** | 15 | Population | COPD patients, aged 55–84 (mean age 72 ± 9 years) |
|  | 16 | Inclusion/Exclusion Criteria | COPD patients with recent exacerbations, no VR experience |
|  | 17 | Baseline Characteristics | Varied COPD severity, 44% male |
| **Outcomes** | 18 | Primary Outcomes | High acceptability and usability of the VR-based system |
|  | 19 | Secondary Outcomes | Improved motivation, focus on exercise content, and engagement |
|  | 20 | Effectiveness Results | Increased motivation and engagement due to the novel, immersive approach |
|  | 21 | Behavioral Outcomes | Positive feedback on ease of use and enjoyment of the exercises |
|  | 22 | Engagement Metrics | High engagement, increased focus during exercises, minimal distractions |
|  | 23 | Quality of Life Improvements | NA |
| **Study Quality and Bias** | 24 | Risk of Bias Assessment | NA |
|  | 25 | Risk of Bias Findings | Low risk as most participants completed the tasks easily |
|  | 26 | Limitations | Small sample size, short duration, lack of long-term follow-up |
| **Adaptation** | 27 | Cultural or Demographic Adaptation | NA |
|  | 28 | Technical Adaptation | Simplified interface for older adults with limited tech skills |
|  | 29 | Challenges in Adaptation | Difficulty with headset weight, loading screens, and initial app navigation |
| **Conclusions** | 30 | Study Conclusion | The VR-based system was well-received, enhancing patient engagement and motivation in pulmonary rehabilitation |
|  | 31 | Relevance to Gamified COPD Management | Highly relevant as a gamified, engaging tool for COPD self-management |
|  | 32 | Recommendations for Future Research | Further research on long-term engagement and clinical effectiveness of VR-based PR systems |

#### Appendix 3-24: Pancini et al., 2023

| **Study 24** | | | |
| --- | --- | --- | --- |
| **Section** | **Q#** | **Data Extracted** | **Details** |
| **Study Information** | 1 | Study ID | Pancini et al., 2023 |
|  | 2 | Title | oVeRcomING COPD: Virtual Reality and Savoring to Promote the Well-Being of Patients with Chronic Obstructive Pulmonary Disease |
|  | 3 | Authors | Elisa Pancini, Daniela Villani, Giuseppe Riva |
|  | 4 | Year of Publication | 2023 |
|  | 5 | Journal/Source | *Cyberpsychology, Behavior, and Social Networking* |
| **Study Design** | 6 | Study Type | RCT (planned) |
|  | 7 | Sample Size | NA (study is in planning phase) |
|  | 8 | Study Setting | IRCCS INRCA, Italy |
|  | 9 | Intervention Duration | 2 weeks (four 20-minute sessions) |
| **Intervention Characteristics** | 10 | Type of Gamified Intervention | VR-based relaxation combined with savoring strategies |
|  | 11 | Platform/Technology Used | VR headset with immersive natural scenarios (e.g., beaches, gardens) |
|  | 12 | Game Elements | Narrated virtual walks, visual/audio feedback, positive emotion amplification |
|  | 13 | Tailoring/Personalization | Personalized savoring exercises to enhance positive emotions |
|  | 14 | Integration with Healthcare | Incorporated into standard pulmonary rehabilitation |
| **Participant Characteristics** | 15 | Population | COPD patients (hospitalized for pulmonary rehabilitation) |
|  | 16 | Inclusion/Exclusion Criteria | Inclusion: COPD patients with anxiety, depression, or stress |
|  | 17 | Baseline Characteristics | NA (trial planned) |
| **Outcomes** | 18 | Primary Outcomes | Reduction in anxiety, depression, stress; increased relaxation and emotional well-being |
|  | 19 | Secondary Outcomes | Enhanced positive emotions and psychological well-being |
|  | 20 | Effectiveness Results | Expected to improve emotional well-being (based on prior research with similar methods) |
|  | 21 | Behavioral Outcomes | Participants expected to experience increased emotional resilience |
|  | 22 | Engagement Metrics | High engagement anticipated due to immersive VR and personalized savoring exercises |
|  | 23 | Quality of Life Improvements | Expected improvements in emotional and psychological well-being |
| **Study Quality and Bias** | 24 | Risk of Bias Assessment | NA (planned study) |
|  | 25 | Risk of Bias Findings | NA (study pending implementation) |
|  | 26 | Limitations | Small sample size, short intervention duration |
| **Adaptation** | 27 | Cultural or Demographic Adaptation | NA |
|  | 28 | Technical Adaptation | Simplified interface to ensure ease of use for elderly patients |
|  | 29 | Challenges in Adaptation | None mentioned, but anticipated VR system adjustments based on user feedback |
| **Conclusions** | 30 | Study Conclusion | VR-based savoring can enhance emotional well-being and reduce psychological distress in COPD patients |
|  | 31 | Relevance to Gamified COPD Management | Highly relevant for integrating VR in COPD care to manage mental health and emotional well-being |
|  | 32 | Recommendations for Future Research | Further studies to assess the long-term impact of savoring and VR interventions on psychological outcomes in COPD |

#### Appendix 3-25: Pardos et al., 2023

| **Study 25** | | | |
| --- | --- | --- | --- |
| **Section** | **Q#** | **Data Extracted** | **Details** |
| **Study Information** | 1 | Study ID | Pardos et al., 2023 |
|  | 2 | Title | Enriching Remote Monitoring and Care Platforms with Personalized Recommendations to Enhance Gamification and Coaching |
|  | 3 | Authors | Antonios Pardos, Parisis Gallos, Andreas Menychtas, Christos Panagopoulos, Ilias Maglogiannis |
|  | 4 | Year of Publication | 2023 |
|  | 5 | Journal/Source | *Studies in Health Technology and Informatics* |
| **Study Design** | 6 | Study Type | Methodology and pilot design |
|  | 7 | Sample Size | Pilot system; no specific patient sample provided |
|  | 8 | Study Setting | Remote care platforms for chronic disease management |
|  | 9 | Intervention Duration | N/A |
| **Intervention Characteristics** | 10 | Type of Gamified Intervention | Personalized coaching with exergames and mental health games |
|  | 11 | Platform/Technology Used | Smartphone app, smartwatches, Bluetooth-enabled devices |
|  | 12 | Game Elements | Credit-based system, scores, rewards, and health recommendations |
|  | 13 | Tailoring/Personalization | Customized recommendations based on personal health data and WHO guidelines |
|  | 14 | Integration with Healthcare | Integration with Personal Health Records (PHR) and third-party apps |
| **Participant Characteristics** | 15 | Population | Not specified (focuses on general chronic disease management, including COPD) |
|  | 16 | Inclusion/Exclusion Criteria | NA (methodology-focused study) |
|  | 17 | Baseline Characteristics | NA |
| **Outcomes** | 18 | Primary Outcomes | Development of personalized recommendations based on health data |
|  | 19 | Secondary Outcomes | Scoring mechanism for physical activity, BMI, sleep, mental health, etc. |
|  | 20 | Effectiveness Results | Early results show potential for increased adherence to care plans using personalized recommendations |
|  | 21 | Behavioral Outcomes | Expected improvement in health-related behavior through gamification |
|  | 22 | Engagement Metrics | Scoring system with credits aimed at enhancing patient engagement |
|  | 23 | Quality of Life Improvements | Not explicitly measured, but improved engagement and adherence could lead to better health outcomes |
| **Study Quality and Bias** | 24 | Risk of Bias Assessment | NA at this stage (pilot) |
|  | 25 | Risk of Bias Findings | NA |
|  | 26 | Limitations | Exclusion of factors like nutrition, smoking, drinking; system still in development |
| **Adaptation** | 27 | Cultural or Demographic Adaptation | NA |
|  | 28 | Technical Adaptation | Data from smartwatches and Bluetooth devices integrated for monitoring |
|  | 29 | Challenges in Adaptation | Further development required to expand recommendation domains |
| **Conclusions** | 30 | Study Conclusion | Personalized recommendations and gamification can enhance patient coaching and engagement in remote monitoring systems |
|  | 31 | Relevance to Gamified COPD Management | Relevant for improving patient engagement and adherence in COPD management through gamification and personalized coaching |
|  | 32 | Recommendations for Future Research | Further development of the system and testing across various patient populations, including COPD patients |

#### Appendix 3-26: Colombo et al., 2024

| **Study 26** | | | |
| --- | --- | --- | --- |
| **Section** | **Q#** | **Data Extracted** | **Details** |
| **Study Information** | 1 | Study ID | Colombo et al., 2024 |
|  | 2 | Title | A virtual reality-based endurance training program for COPD patients: acceptability and user experience |
|  | 3 | Authors | Vera Colombo, Marta Mondellini, Alessia Fumagalli, Andrea Aliverti, Marco Sacco |
|  | 4 | Year of Publication | 2024 |
|  | 5 | Journal/Source | *Disability and Rehabilitation: Assistive Technology* |
| **Study Design** | 6 | Study Type | Single-group pilot study |
|  | 7 | Sample Size | N=14 (12 completed the study) |
|  | 8 | Study Setting | In-hospital rehabilitation program, Italy |
|  | 9 | Intervention Duration | Two weeks, with 20-minute sessions, twice daily |
| **Intervention Characteristics** | 10 | Type of Gamified Intervention | Semi-immersive VR cycling in a virtual park environment |
|  | 11 | Platform/Technology Used | Cycle-ergometer, pulse oximeter, wide screen projection |
|  | 12 | Game Elements | Visual feedback, real-time cycling metrics, first-person navigation |
|  | 13 | Tailoring/Personalization | Exercise intensity based on baseline conditions |
|  | 14 | Integration with Healthcare | Continuous supervision by physiotherapists |
| **Participant Characteristics** | 15 | Population | 6 females, 8 males, average age 71.29 years with mild to moderate COPD |
|  | 16 | Inclusion/Exclusion Criteria | Included patients with stable COPD; excluded those with severe comorbidities or impairments |
|  | 17 | Baseline Characteristics | 6MWT score of 478.00 ± 80.44 meters |
| **Outcomes** | 18 | Primary Outcomes | Adherence rate of 85.71%, 6MWT improved to 520.50 ± 69.24 meters |
|  | 19 | Secondary Outcomes | High user engagement (Short Flow State Scale 4.40 ± 0.36), fatigue and dyspnea improvements |
|  | 20 | Effectiveness Results | Significant improvements in exercise capacity (*p*<0.05) |
|  | 21 | Behavioral Outcomes | Increased motivation to exercise through VR |
|  | 22 | Engagement Metrics | 86.85% attendance rate |
|  | 23 | Quality of Life Improvements | No specific data reported for SGRQ/SF-36 |
| **Study Quality and Bias** | 24 | Risk of Bias Assessment | NA |
|  | 25 | Risk of Bias Findings | Low risk of bias based on high adherence and positive outcomes |
|  | 26 | Limitations | Small sample size, no control group |
| **Adaptation** | 27 | Cultural or Demographic Adaptation | Focused on older Italian patients with COPD |
|  | 28 | Technical Adaptation | Use of semi-immersive VR to suit hospital settings |
|  | 29 | Challenges in Adaptation | Issues with scaling workload increments |
| **Conclusions** | 30 | Study Conclusion | VR endurance training is feasible and well-accepted by COPD patients |
|  | 31 | Relevance to Gamified COPD Management | Supports the use of VR for engagement and adherence in pulmonary rehabilitation |
|  | 32 | Recommendations for Future Research | Suggests larger studies with control groups and long-term assessment |

#### Appendix 3-27: Jin et al., 2024

| **Study 27** | | | |
| --- | --- | --- | --- |
| **Section** | **Q#** | **Data Extracted** | **Details** |
| **Study Information** | 1 | Study ID | Jin et al., 2024 |
|  | 2 | Title | The Association of Conventional Therapy Associated with Somatosensory Interactive Game Enhances the Effects of Early Pulmonary Rehabilitation for Patients with AECOPD |
|  | 3 | Authors | Xiaoliang Jin, Mengni Jin, Beilei Zhang, Mei’e Niu, Yanxia Han, Jiale Qian |
|  | 4 | Year of Publication | 2024 |
|  | 5 | Journal/Source | *Games for Health Journal* |
| **Study Design** | 6 | Study Type | Randomized Controlled Trial (RCT) |
|  | 7 | Sample Size | N=80 (EG: 40, CG: 40) |
|  | 8 | Study Setting | The First Affiliated Hospital of Soochow University, China |
|  | 9 | Intervention Duration | 6 weeks (daily 20-min interactive game + 30-min conventional therapy) |
| **Intervention Characteristics** | 10 | Type of Gamified Intervention | Somatosensory interactive games involving arm movements for exercise |
|  | 11 | Platform/Technology Used | Motion-based games: “Kitchen Sharp Knife,” “Swimming Master,” “Table Tennis Master” |
|  | 12 | Game Elements | Real-time visual feedback, engaging tasks |
|  | 13 | Tailoring/Personalization | Patients adjusted exercise based on comfort level |
|  | 14 | Integration with Healthcare | Integrated with physiotherapist-supervised pulmonary rehabilitation programs |
| **Participant Characteristics** | 15 | Population | 73.97 years average age, AECOPD patients |
|  | 16 | Inclusion/Exclusion Criteria | AECOPD diagnosis, physical ability to perform the intervention |
|  | 17 | Baseline Characteristics | 6MWD: 300.64 ± 81.92 meters, FEV1: 1.22 L, FVC: 2.41 L |
| **Outcomes** | 18 | Primary Outcomes | Significant improvements in 6MWD and Brief-BESTest at 3 months post-intervention (*p*<0.001) |
|  | 19 | Secondary Outcomes | No significant change in inflammatory markers (CRP, PCT) or healthcare utilization |
|  | 20 | Effectiveness Results | Experimental group maintained higher endurance and balance for 12 months |
|  | 21 | Behavioral Outcomes | Enhanced exercise tolerance and balance function, motivation sustained for 3 months |
|  | 22 | Engagement Metrics | 82.5% adherence in the intervention group |
|  | 23 | Quality of Life Improvements | Significant balance and exercise tolerance improvement |
| **Study Quality and Bias** | 24 | Risk of Bias Assessment | NA |
|  | 25 | Risk of Bias Findings | Low, based on group similarity and controlled environment |
|  | 26 | Limitations | Lack of long-term adherence tracking, reliance on self-report |
| **Adaptation** | 27 | Cultural or Demographic Adaptation | Tailored to older Chinese COPD patients |
|  | 28 | Technical Adaptation | Visual feedback and game variety catered to balance and respiratory issues |
|  | 29 | Challenges in Adaptation | Unclear measurement of exercise intensity |
| **Conclusions** | 30 | Study Conclusion | SIG with PRP enhanced exercise and balance function, sustaining effects for 12 months |
|  | 31 | Relevance to Gamified COPD Management | High relevance due to the use of gamification to enhance pulmonary rehabilitation outcomes |
|  | 32 | Recommendations for Future Research | Need for studies on long-term effects and immune response markers |

#### Appendix 3-28: Kizmaz et al., 2024

| **Study 28** | | | |
| --- | --- | --- | --- |
| **ection** | **Q#** | **Data Extracted** | **Details** |
| **Study Information** | 1 | Study ID | Kizmaz et al., 2024 |
|  | 2 | Title | Virtual reality for COPD exacerbation: A randomized controlled trial |
|  | 3 | Authors | Erhan Kizmaz, Orcin Telli Atalay, Nazlı Çetin, Erhan Uğurlu |
|  | 4 | Year of Publication | 2024 |
|  | 5 | Journal/Source | *Respiratory Medicine* |
| **Study Design** | 6 | Study Type | RCT |
|  | 7 | Sample Size | N=50 |
|  | 8 | Study Setting | Hospitalized patients at Pamukkale University, Turkey |
|  | 9 | Intervention Duration | Until discharge from hospitalization |
| **Intervention Characteristics** | 10 | Type of Gamified Intervention | Virtual reality cycling simulation in the forest combined with PR |
|  | 11 | Platform/Technology Used | Oculus Quest 2 VR headset |
|  | 12 | Game Elements | Immersive cycling simulation in a forest environment |
|  | 13 | Tailoring/Personalization | Not reported |
|  | 14 | Integration with Healthcare | Integrated with PR sessions, supervised by physiotherapists |
| **Participant Characteristics** | 15 | Population | COPD patients aged 35-85 hospitalized for exacerbation |
|  | 16 | Inclusion/Exclusion Criteria | Inclusion: FEV1 < 70%, FEV1/FVC < 70%, Hodkinson Mental Test score >6; Exclusion: respiratory diseases more severe than COPD, heart failure, recent surgeries, etc. |
|  | 17 | Baseline Characteristics | Mean age: PR + VR group 62.64 years, PR group 64.24 years; similar characteristics in height, weight, and BMI |
| **Outcomes** | 18 | Primary Outcomes | STST: significant improvement in PR + VR group (*p* < 0.001); CAT: significant reduction (*p* < 0.001) |
|  | 19 | Secondary Outcomes | HADS: greater reduction in PR + VR group (*p* < 0.001); Dyspnea levels (mMRC) reduced significantly |
|  | 20 | Effectiveness Results | VR + PR group had greater improvements in functional capacity, symptom reduction, anxiety, depression, and daily living activities compared to PR alone |
|  | 21 | Behavioral Outcomes | Increased motivation and adherence to exercise reported in VR + PR group |
|  | 22 | Engagement Metrics | VR + PR group had a significantly longer pedaling time (508.44s vs. 357.56s, *p* = 0.007) |
|  | 23 | Quality of Life Improvements | Greater improvement in daily activities (LCADL) in PR + VR group (*p* < 0.001) |
| **Study Quality and Bias** | 24 | Risk of Bias Assessment | NA |
|  | 25 | Risk of Bias Findings | Likely low risk, given the blinded evaluator and randomized design |
|  | 26 | Limitations | No objective assessment of cyber-sickness or patient satisfaction; no third group for comparison with usual care |
| **Adaptation** | 27 | Cultural or Demographic Adaptation | NA |
|  | 28 | Technical Adaptation | Real-world footage of cycling in a forest used to enhance ecological realism |
|  | 29 | Challenges in Adaptation | One patient could not continue due to dizziness related to VR use |
| **Conclusions** | 30 | Study Conclusion | VR combined with PR contributes significantly to improving functional capacity, symptoms, and mental health during COPD exacerbation |
|  | 31 | Relevance to Gamified COPD Management | Highly relevant for integrating VR into COPD exacerbation management to increase motivation and exercise adherence |
|  | 32 | Recommendations for Future Research | Future studies should assess patient satisfaction and evaluate long-term outcomes and cyber-sickness |

#### Appendix 3-29: McAnirlin et al., 2024

| **Study 29** | | | |
| --- | --- | --- | --- |
| **Section** | **Q#** | **Data Extracted** | **Details** |
| **Study Information** | 1 | Study ID | McAnirlin et al., 2024 |
|  | 2 | Title | Co-creating and delivering personalized, nature-based VR experiences: Proof-of-concept study with four U.S. adults living with severe COPD |
|  | 3 | Authors | McAnirlin, Browning, Fasolino, Okamoto, Sharaievska, Thrift, Pope |
|  | 4 | Year of Publication | 2024 |
|  | 5 | Journal/Source | *Wellbeing, Space and Society* |
| **Study Design** | 6 | Study Type | Proof-of-concept, mixed methods |
|  | 7 | Sample Size | N=4 |
|  | 8 | Study Setting | Upstate South Carolina, U.S.; participants’ homes |
|  | 9 | Intervention Duration | May-August 2022 |
| **Intervention Characteristics** | 10 | Type of Gamified Intervention | Nature-based virtual reality experiences |
|  | 11 | Platform/Technology Used | Oculus Quest 2 headset |
|  | 12 | Game Elements | Co-created 360-degree videos of personalized, nature-based scenes |
|  | 13 | Tailoring/Personalization | Personalized VR based on participants’ outdoor memories |
|  | 14 | Integration with Healthcare | Not integrated; exploratory focus on well-being |
| **Participant Characteristics** | 15 | Population | Adults (55-73 years old) with severe COPD |
|  | 16 | Inclusion/Exclusion Criteria | Inclusion: Severe COPD, older adults; Exclusion: Recent myocardial infarction, motion sickness |
|  | 17 | Baseline Characteristics | All on oxygen therapy, with variation in caregiver support |
| **Outcomes** | 18 | Primary Outcomes | PWB, HR, RR, SPO2 |
|  | 19 | Secondary Outcomes | Presence, restoration, cybersickness, oral feedback |
|  | 20 | Effectiveness Results | Positive changes in well-being and presence, no cybersickness reported |
|  | 21 | Behavioral Outcomes | Participants experienced positive emotional responses, reflective of nostalgic memories |
|  | 22 | Engagement Metrics | Participants co-created their own VR experiences, leading to high engagement and satisfaction |
|  | 23 | Quality of Life Improvements | Reported feelings of autonomy, positive emotions linked to memories, restorative effects |
| **Study Quality and Bias** | 24 | Risk of Bias Assessment | NA |
|  | 25 | Risk of Bias Findings | NA |
|  | 26 | Limitations | Small sample size, no control group, exploratory design |
| **Adaptation** | 27 | Cultural or Demographic Adaptation | Customized to individual preferences and memories |
|  | 28 | Technical Adaptation | Personalized VR experiences were created using 360-degree videos |
|  | 29 | Challenges in Adaptation | Customization required multiple visits and effort to personalize scenes |
| **Conclusions** | 30 | Study Conclusion | Co-created VR experiences show potential for enhancing well-being in people with severe COPD |
|  | 31 | Relevance to Gamified COPD Management | Offers insights into using personalized VR to support psychological health in severe COPD |
|  | 32 | Recommendations for Future Research | Explore scalability, long-term impact of co-created VR in COPD patients, and expand sample size |
